# Protein sensing in living cells by molecular rotor-based fluorescence-switchable chemical probes†
†Electronic supplementary information (ESI) available: See DOI: 10.1039/c5sc02808f



**DOI:** 10.1039/c5sc02808f

**Published:** 2015-10-01

**Authors:** Wan-Ting Yu, Ting-Wei Wu, Chi-Ling Huang, I-Chia Chen, Kui-Thong Tan

**Affiliations:** a Department of Chemistry , National Tsing Hua University , 101 Sec. 2, Kuang Fu Rd , Hsinchu 30013 , Taiwan , Republic of China . Email: kttan@mx.nthu.edu.tw ; Tel: +886-3-5715131; b Frontier Research Center on Fundamental and Applied Sciences of Matters , National Tsing Hua University , 101 Sec. 2, Kuang Fu Rd , Hsinchu 30013 , Taiwan , Republic of China

## Abstract

In this paper, we introduce a general design to construct fluorescence-switching probes by using conjugates of a fluorescent molecular rotor and protein specific ligands for the selective protein detection and real-time tracking of protein degradation in living cells. Upon the interaction of the ligand with the protein ligand-binding domain, the crowded surroundings restrict the bond rotation of the fluorescent molecular rotor to trigger the emission of a strong fluorescence signal, which is reduced upon the addition of a competitive ligand or after protein degradation. With this probe design, two fluorescent probes for MGMT and hCAII proteins were constructed and applied for detecting the endogenous proteins in living cells. In addition, real-time degradation kinetics of the alkylated-MGMT at the single living cell level were revealed for the first time. We believe that this fluorescence-switching probe design can possibly be extended for the analysis of other proteins, for which there are still no effective tools to visualize them in living cells.

## Abstract 2

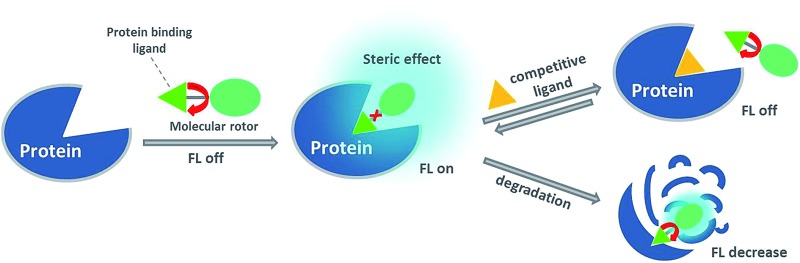
We introduce a general design to construct fluorescence-switching probes. Upon the interaction of the ligand with the protein, the crowded surroundings restrict the bond rotation of the fluorescent molecular rotor to trigger a strong fluorescence signal, which is reduced upon the addition of a competitive ligand or after protein degradation.

## Introduction

Fluorescent chemical probes that respond to the protein expression level, activity and degradation in living cells are important tools in biology to study cellular processes because they allow for sensitive, simple and specific analysis of target proteins.^1,2^ Most fluorescent probes have been designed for monitoring the activity of enzymes, such as glycosidases,^3^ proteases,^4^ phosphatases^5^ and nitroreductase.^6,7^ Typically, these enzymes react with the recognition groups on the fluorescent probes and subsequently, a fluorescence signal can be switched-on *via* several fluorescence activation mechanisms, such as FRET, PET and the restoration of fluorophore π-conjugation. Although undoubtedly valuable and widely described in the literature, there are major limitations that restrict the use of these fluorescent probes in protein analysis. For example, this probe design is not applicable to non-enzymatic proteins which do not possess the necessary enzymatic action to disrupt the probe structure. Furthermore, fluorescence activation of these fluorescent probes is an irreversible process, which confines their application to the detection of the protein expression level and activity. In contrast, protein-specific fluorescence-switchable probes exhibiting reversible and multiple fluorescence off/on cycles would present a more desirable feature that can be applied to detect the protein expression level and activity, and also subsequent analysis of protein degradation, translocation and inhibitor screening in cells.^8,9^

There are many obstacles that need to be addressed in order to construct operative fluorescence-switchable probes for protein sensing and analysis inside living cells, such as a high cell permeability, effective fluorescence-switching functions and a high protein selectivity. To date, the development of this type of fluorescent probe remains a challenging task and only a limited number of fluorescence-switching strategies have been reported. In most of the designs, fluorescence-switchable probes were constructed by conjugating a protein binding ligand with various fluorescence-switchable fluorophores, such as solvatochromic fluorophores,^10–12^ disassembly-induced emission^13–15^ and aggregation-induced emission dyes.^16–18^ However, due to low to moderate fluorescence enhancement ratios and potential unspecific binding of the fluorescent probes to other intracellular proteins, only a few of them have been demonstrated in sensing intracellular proteins. In addition, the fates of the proteins, such as protein degradation, have never been investigated before using this type of fluorescence-switching probe. Clearly, the establishment of a new general strategy to construct fluorescence-switchable probes is highly desirable.

In this paper, we introduce a modular and versatile design to construct fluorescence-switching probes by using conjugates of a fluorescent molecular rotor, 9-(2-carboxy-2-cyanovinyl)julolidine (CCVJ), and protein specific ligands for the selective fluorescence turn-on detection of proteins followed by real-time tracking of protein degradation in living cells. Typically, the emission of CCVJ is characterized by a charge-transfer excited state, which can be rapidly deactivated through intramolecular rotation about the donor–acceptor bond. In a highly constrained environment, such as in glycerol solution or upon binding with proteins, a large fluorescence increase can be observed due to the restricted bond rotation of the fluorophore.^19–21^ Because of their emission properties, CCVJ and its derivatives have since been employed as probes for local viscosity as well as for sensing free volume and plasticity in polymers.^22–25^

In our design, we envisaged that the binding of the ligand to the target protein would bring the molecular rotor CCVJ closer to the crowded protein surrounding. As a result, bond rotation of CCVJ should be sufficiently restricted to trigger the emission of a strong fluorescence signal. In the presence of a competitive ligand or after protein degradation, the fluorescent probe should be ejected from the crowded protein environment and therefore it should exhibit only a weak emission. Although the CCVJ dye has recently been employed to construct fluorescent probes for protein detection, the mere 2-fold fluorescence enhancement in the presence of the target protein limits the application of these probes for the detection of proteins in living cells.^26^

## Results and discussion

### Characterization of fluorescent probe **BG-CCVJ** for the detection of MGMT

To test the fluorescence-switching probe design, O^6^-methylguanine-methyltransferase (MGMT) was chosen as a target protein. MGMT is a suicide enzyme that repairs O^6^-alkylguanine lesions in cells to attenuate the therapeutic effects of many antitumor DNA alkylating agents.^27,28^ After reaction with O^6^-alkylguanine, the alkylated-MGMT will be degraded rapidly in cells. The MGMT activity is highly variable in different human tissues and in the same tissues of different individuals.^29^ Certain human tumor tissues, such as breast and lung malignant tissues, often express more MGMT than adjacent normal tissues. In patients with malignant gliomas, high levels of MGMT activity have been associated with resistance to alkylation drugs, leading to treatment failure.^30,31^ Therefore, MGMT levels in tumor cells have been proposed as a prognostic marker for tumor resistance to O^6^-alkylating agents, which would provide a guide for therapeutic decisions.^32^ Although several methods have been developed to determine cellular MGMT levels, including the use of radioisotope-labeled O^6^-benzylguanine pseudosubstrates,^33^ immunoassays^34^ and promoter methylation specific PCR,^35^ all of them are complex, laborious and time-consuming. For MGMT activity detection, common enzymatic fluorescent probe designs are not applicable. This is because dealkylation of O^6^-alkylguanine by MGMT does not directly affect the fluorescence properties of the fluorophore, which is connected to the enzyme-cleavable moiety in the probe. So far, a fluorescent probe that can undergo a large fluorescence enhancement as a result of the reaction between MGMT and the probe is still not available.

**Fig. 1:**
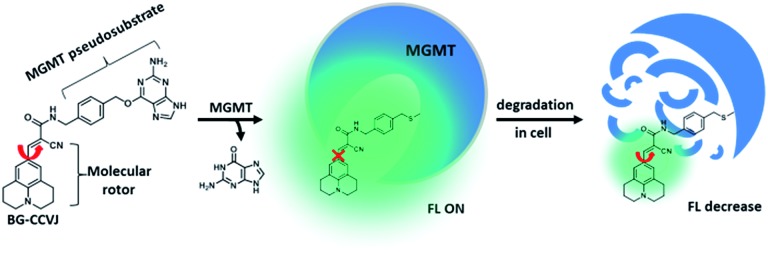
Schematic illustration of the fluorescence-switchable probe **BG-CCVJ** for the detection of MGMT activity and real-time tracking of alkylated-MGMT degradation in living cells.

Based on the concept of our fluorescent probe design, we developed a MGMT-activated fluorescence turn-on probe, **BG-CCVJ**, which consists of a specific MGMT suicide pseudosubstrate, O^6^-benzylguanine (O^6^-BG) and a fluorescent molecular rotor, CCVJ (Fig. 1). In the presence of MGMT, the enzyme transfers the CCVJ fragment to the protein active site where the crowded surroundings restrict the bond rotation of the fluorescent molecular rotor to trigger the emission of a strong fluorescence signal, which is reduced upon CCVJ-labeled MGMT degradation. The probe **BG-CCVJ** can be prepared in a single step by reacting CCVJ with BG-NH_2_ under standard peptide coupling conditions in 86% yield after purification (Scheme S1†).

**Fig. 2:**
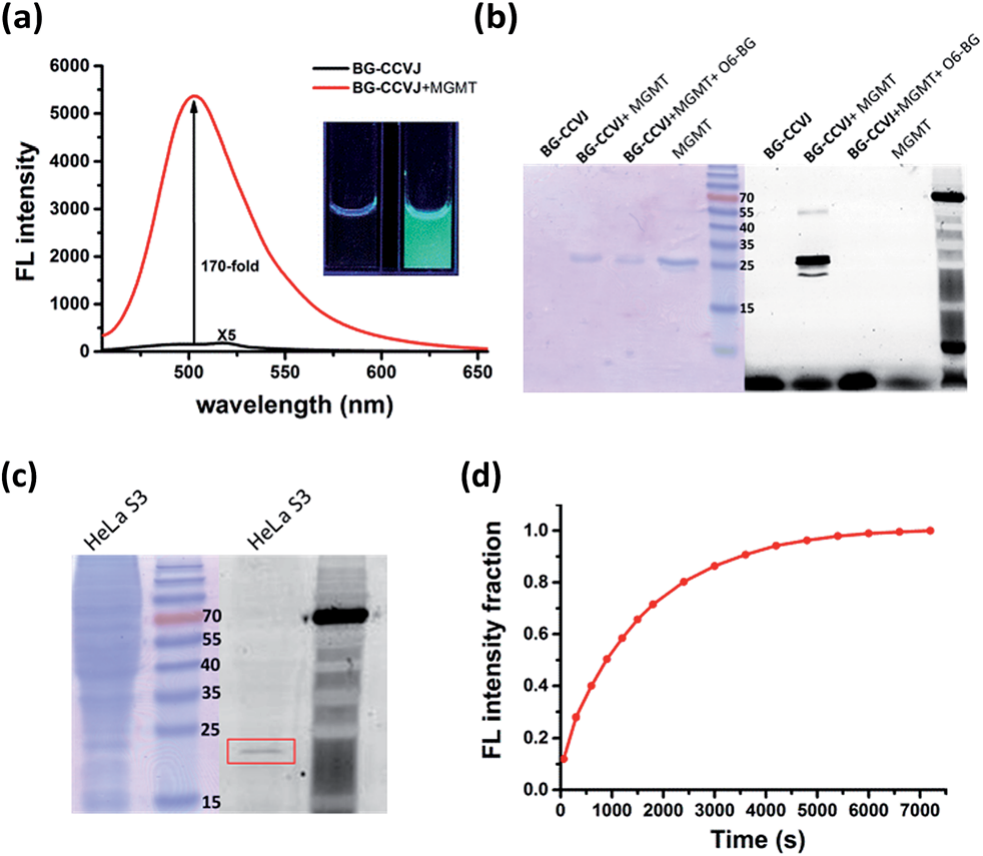
Characterization of the fluorescence activation probe **BG-CCVJ** for the detection of MGMT. (a) Fluorescence spectra of 5 μM **BG-CCVJ** in the absence and presence of 5 μM MGMT in PBS buffer (1% DMSO). The fluorescence spectrum of free **BG-CCVJ** is magnified 5-times. *λ*_ex_ = 440 nm. The inset shows the images of the solution in a cuvette before (left) and after (right) the addition of 5 μM MGMT under excitation with an UV lamp (365 nm). (b) Covalent reaction of **BG-CCVJ** and recombinant MGMT studied by SDS-PAGE gel. The gel was fluorescently scanned (right), followed by staining with Instant Blue (left). The molecular weight of the recombinant MGMT is about 26 kDa. (c) Selective reaction of 5 μM **BG-CCVJ** with HeLa S3 cell lysates analyzed by SDS-PAGE gel. The gel was fluorescently scanned (right), followed by staining with Instant Blue (left). The alkylated native MGMT (MW of about 23 kDa) is highlighted in the red rectangular box. (d) Time course of the **BG-CCVJ** reaction with MGMT. Reaction conditions: 5 μM **BG-CCVJ** was mixed with 5 μM MGMT in a 96-well plate. The emission was measured immediately after mixing by monitoring the fluorescence increase at 504 nm.

In aqueous buffer, **BG-CCVJ** shows an extremely weak fluorescence signal (Fig. 2a). However, the fluorescence signal was enhanced dramatically in the presence of the MGMT protein with a high activation ratio of around 170-fold. The transfer of CCVJ to MGMT induced a blue-shift of the maximum emission wavelength of **BG-CCVJ** from 518 nm in PBS buffer to 504 nm when MGMT was added. The fluorescence enhancement was so significant that it can be observed easily under a hand-held UV lamp (Fig. 2a, inset). A Job's plot analysis was performed to determine the stoichiometry of the complex formed between **BG-CCVJ** and MGMT. The fluorescence intensity of **BG-CCVJ** peaked at 1 : 1 mole fraction of **BG-CCVJ** to MGMT, which indicates that the probe binds mainly at the BG binding site of the protein (Fig. S1†). This dramatic fluorescence increase can be obtained in a wide physiological pH range within pH 4–10 (Fig. S2†). SDS-PAGE gel fluorometric analysis confirmed that the dramatic fluorescence increase was due to the transfer of the CCVJ group to MGMT to form a covalent bond conjugate (Fig. 2b). A fluorescence band was observed only when **BG-CCVJ** was mixed with the MGMT protein, and was not visible in the presence of the MGMT inhibitor (O^6^-BG, 100 μM). When MGMT was added in increasing concentrations, the probe showed a concentration dependent fluorescence enhancement (Fig. S3†). With 5 μM **BG-CCVJ**, the limit of detection (LOD) to detect MGMT was determined to be as low as 5 nM.

Besides the superior sensitivity and an extremely high fluorescence enhancement, we also tested the reaction of **BG-CCVJ** with MGMT in cell lysates. When **BG-CCVJ** was incubated with the lysate of HeLa S3 cells, which express a substantial amount of MGMT, only a single fluorescence band with a molecular weight of around 23 kDa was observed (Fig. 2c). Furthermore, we also studied the selectivity of **BG-CCVJ** against a collection of eleven proteins (Fig. S4†). In this selectivity test, a very weak fluorescence signal was observed when **BG-CCVJ** was mixed with non-BG recognition proteins. In contrast, a strong fluorescence enhancement was obtained when MGMT was incubated with **BG-CCVJ**. These results show that the fluorescence activation is controlled by the selective recognition of MGMT to the O^6^-BG group of **BG-CCVJ**.

A kinetic analysis of the **BG-CCVJ** reaction with MGMT was carried out by continuously monitoring the increase in the fluorescence intensity in a microtiter plate at protein and probe concentrations of 5 μM each (Fig. 2d). The time required to achieve full labeling of MGMT was approximately 6500 seconds. The second-order rate constant (*k*_2_) for the reaction between **BG-CCVJ** and MGMT was determined to be about 715 M^–1^ s^–1^ (Fig. S5†). The *k*_2_ value for the reaction of MGMT with our **BG-CCVJ** probe is similar to the reported *k*_2_ value of about 600 M^–1^ s^–1^ obtained using the radioisotope-labeled O^6^-BG substrate.^36^ This indicates that the introduction of the CCVJ fluorescent molecular rotor to the O^6^-BG moiety does not alter the reaction kinetics, and **BG-CCVJ** can be an alternative and convenient probe to radioisotope-labeled substrates to monitor MGMT activity.

### Fluorescence activation mechanism of **BG-CCVJ** in the presence of MGMT

**Fig. 3:**
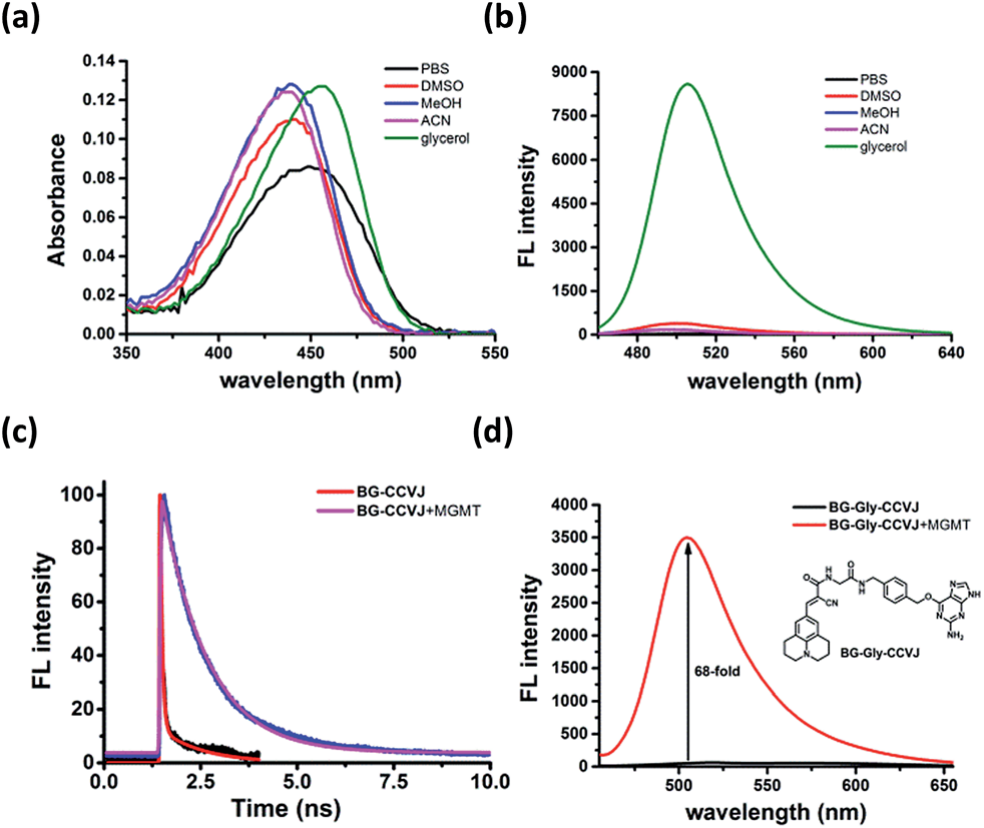
Characterization of the **BG-CCVJ** fluorescence activation mechanism in the presence of MGMT. (a) Absorption and (b) emission spectra of the **BG-CCVJ** probe (5 μM) in DMSO, MeOH, ACN, PBS and glycerol. (c) Fluorescence lifetime of **BG-CCVJ** in the absence and presence of MGMT. (d) Fluorescence responses of 5 μM **BG-Gly-CCVJ** in the absence and presence of 5 μM MGMT. The inset shows the chemical structure of **BG-Gly-CCVJ**.

To ascertain that the fluorescence increase of **BG-CCVJ** is due to the restricted rotation of the fluorescent molecular rotor upon transferring CCVJ to MGMT, we investigated the absorption and emission spectra of the **BG-CCVJ** probe in various solvents (DMSO, MeOH, ACN, PBS and glycerol) with different polarities and viscosities. While the absorption spectra of **BG-CCVJ** do not show much difference in these solvents (Fig. 3a), a strong emission can be observed only in the highly viscous glycerol solution (Fig. 3b). In glycerol, the maximum emission of **BG-CCVJ** is at 505 nm, which is similar to that when CCVJ is located at the BG binding pocket of MGMT. In addition, the fluorescence lifetime of **BG-CCVJ** in the absence or presence of MGMT was also investigated (Fig. 3c). In the absence of MGMT, **BG-CCVJ** exhibited a bi-exponential fluorescence decay with lifetimes (*t*) of 0.05 ns and 1.12 ns. In the presence of MGMT, the probe displayed a longer single exponential fluorescence decay with a lifetime of 1.16 ns. Together, these results are consistent with the characteristics of fluorescent molecular rotors, which show longer fluorescence lifetimes and stronger emissions in a more restricted environment. Furthermore, we also synthesized a **BG-CCVJ** derivative by incorporating an amino acid glycine as a linker in between O^6^-BG and the CCVJ dye to form **BG-Gly-CCVJ**. In the presence of MGMT, **BG-Gly-CCVJ** exhibited a 68-fold increase in the fluorescence signal, which is about 2.5-fold lower than that observed in the reaction of **BG-CCVJ** with MGMT (Fig. 3d). The smaller fluorescence increase obtained from **BG-Gly-CCVJ** suggests that the fluorescence enhancement is steric-dependent and the optimal result can be obtained when the fluorescent molecular rotor is closer to the protein binding pocket, which imposes a higher steric hindrance to the intra-molecular bond rotation of the dye.

### Rapid live-cell imaging of MGMT activities with **BG-CCVJ**

**Fig. 4:**
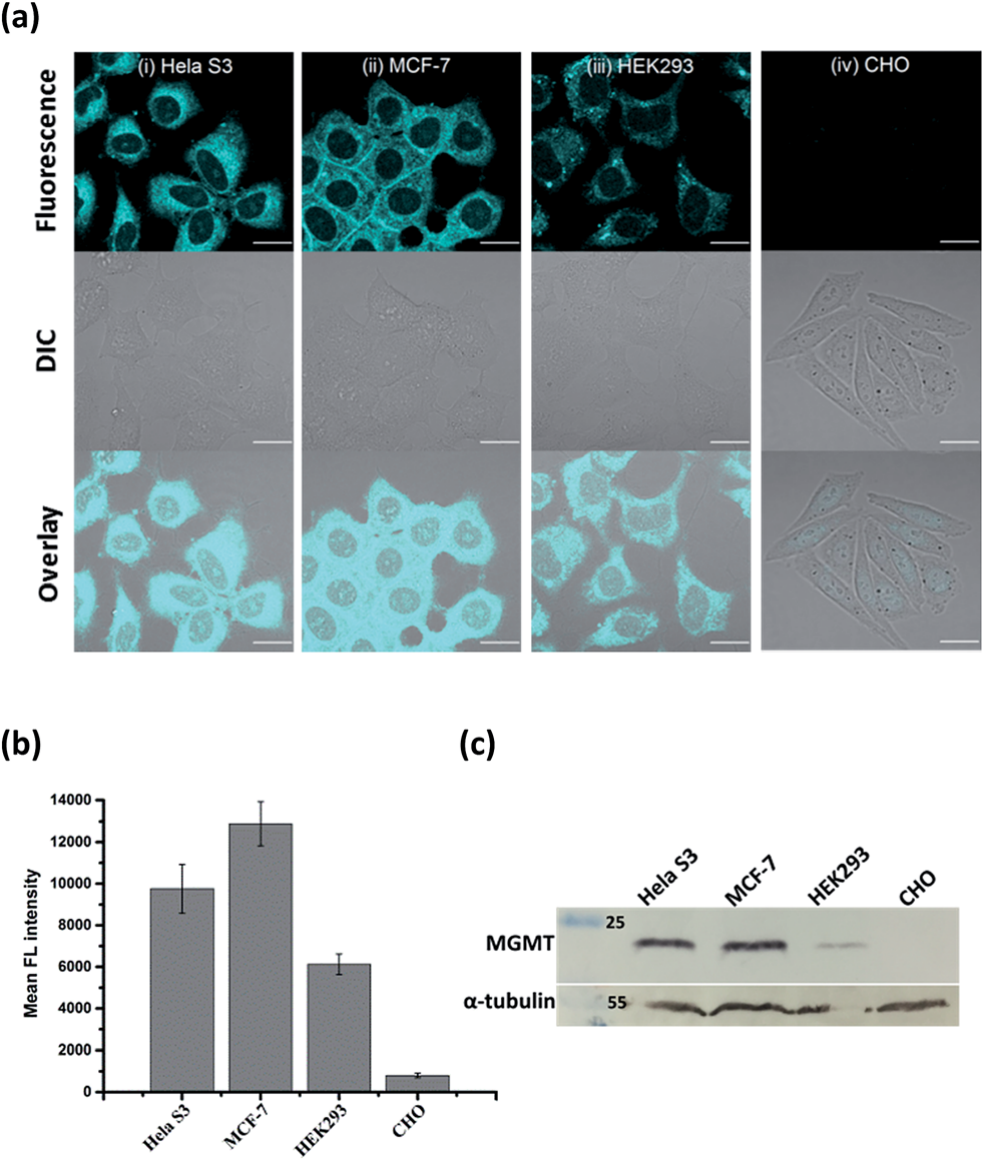
No-wash live-cell imaging of the MGMT activities with **BG-CCVJ**. (a) Images of live HeLa S3, MCF-7, HEK293 and CHO cells treated with 1 μM **BG-CCVJ**. Images were taken immediately without any washing procedures after 90 minutes of incubation with **BG-CCVJ**. All cellular images were taken with an identical microscope setup. Scale bar: 20 μm. (b) Mean fluorescence intensity of the cells treated with **BG-CCVJ** (*N* = 20). (c) Western blot analysis of the MGMT levels in HeLa S3, MCF-7, HEK293 and CHO cells with the anti-MGMT antibody. α-Tubulin in the cells was detected with the anti-α-tubulin antibody and was used as the positive control.

Since the fluorescence of **BG-CCVJ** is activated only when it is covalently bound to MGMT, the low background signal of the probe should allow us to image the MGMT activity in cells without the washing operation so that high-throughput imaging of a large number of tumor cells can be rapidly achieved to differentiate those that express variable amounts of MGMT. Four different cell lines, HeLa S3, MCF-7, HEK293 and CHO cells, were selected to demonstrate the application of no-wash live-cell imaging of MGMT activities with **BG-CCVJ**. Apart from CHO cells, which are MGMT-deficient, the other three types of cell lines have been known to express substantial amounts of MGMT.^33,37^ Live-cell images were taken immediately without any washout process after 1 μM **BG-CCVJ** was incubated with the cells for 90 minutes (Fig. S6†). A strong fluorescence signal in the cytosol was observed for the HeLa S3, MCF-7, and HEK293 cells, while the CHO cells showed a very weak fluorescence signal when using a confocal laser scanning microscope (Fig. 4a). Prior investigations using fractional cell extracts and immunostaining showed that MGMT in HeLa S3 cells is mostly localized in the cytosol.^38,39^ Extensive washing of the cells with medium did not change the imaging results (Fig. S7†). To validate that the fluorescence signals observed from the three MGMT-positive cells are specific and due to the reaction of **BG-CCVJ** with MGMT, we synthesized two negative-control compounds containing no O^6^-BG moiety on the CCVJ fluorophore (Fig. S8†). All the four cell lines used in our studies did not show fluorescence signals upon treatment with these compounds. Although the CCVJ dye exhibits a strong fluorescence signal in a highly viscous environment, the results from the two negative control compounds indicate that an intracellular environment might not be viscous enough to generate a strong fluorescence signal, as in the case of the reaction between **BG-CCVJ** and MGMT.

The level of MGMT in the cells was subsequently quantified by measuring the mean fluorescence intensity of the cells pixel-by-pixel using ImageJ software (Fig. 4b). The results showed that HeLa S3, MCF-7 and HEK293 cells express at least 10- to 20-fold more MGMT protein than CHO cells, with HeLa S3 and MCF-7 cells expressing about 1.5 to 2.5-fold more MGMT than HEK293 cells. The values are in agreement with the previous result obtained using radioisotope-labeled O^6^-BG.^33^ We also performed Western blot analysis to validate the MGMT levels in the four cell lines using the monoclonal anti-MGMT antibody (Fig. 4c). Results from the Western blot analysis correlated very well with the fluorescence images obtained using our **BG-CCVJ** probe – the MGMT-positive cells, *i.e.* the HeLa S3, MCF-7, and HEK293 cells, gave significant Western blot bands, while no obvious band was observed for the CHO cells. Attempts to detect MGMT proteins in cell lysates with **BG-CCVJ** were also conducted. The same conclusions as the imaging experiments were obtained, where HeLa S3, MCF-7, and HEK293 cell lysates showed higher fluorescence intensities than the lysates of the CHO cells (Fig. S9†).

### Real-time tracking of alkylated-MGMT degradation in living cells

Many studies have found that alkylation of MGMT by O^6^-BG substrates or DNA alkylating drug temozolomide leads to rapid degradation of the protein in cells. Previously, experiments to determine the degradation lifetime of alkylated-MGMT were performed by taking aliquots of the cell lysate at distinct time points to be analyzed *via* Western blot analysis or fluorescence scanning of SDS-PAGE gels. However, the ability to monitor the real-time degradation status of alkylated-MGMT at the single cell level would be extremely valuable to understand the degradation mechanism in greater detail. As our **BG-CCVJ** probe exhibits fluorescence-switching properties depending on whether it is in the free form or covalently bound to MGMT state, it can thus be a very useful tool to monitor the real-time degradation status of alkylated-MGMT at the single cell level.

**Fig. 5:**
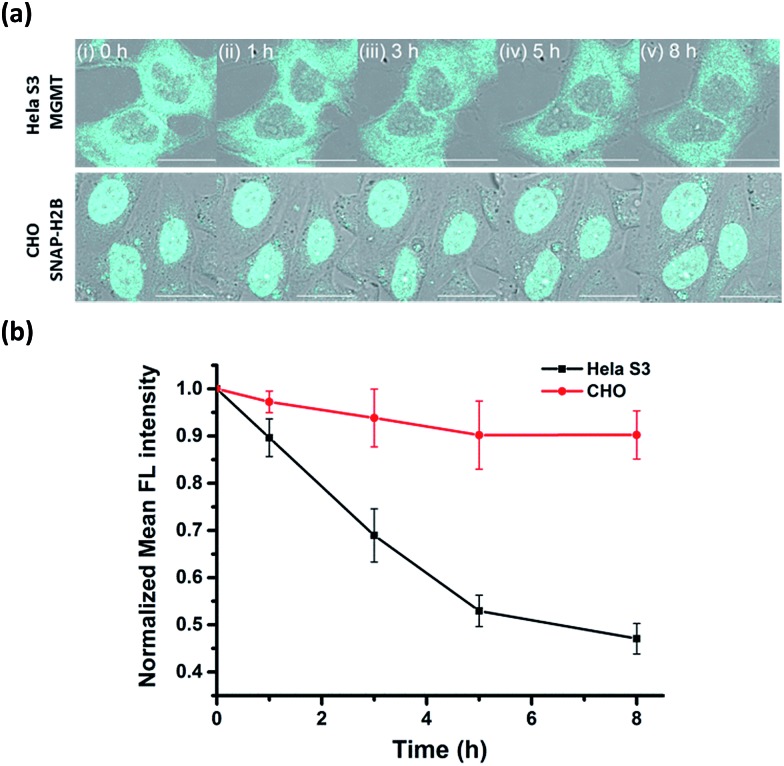
Real-time tracking of alkylated-MGMT degradation in living cells. (a) Fluorescence and DIC overlaid images of HeLa S3 and CHO cells (transfected with SNAP-H2B plasmid) taken at the indicated times. The cells were treated with 50 μM **BG-CCVJ** for ten minutes, followed by extensive washing to remove the unreacted probe. Scale bar: 20 μm. (b) Time course of the mean fluorescence intensity of the HeLa S3 and CHO cells treated with **BG-CCVJ** (*N* = 15).

Real-time tracking of the alkylated-MGMT degradation status was conducted by incubating HeLa S3 cells with 50 μM **BG-CCVJ** for 10 minutes, followed by extensive washing to remove the unreacted probe. The fluorescence of the alkylated-MGMT at the single cell level was monitored by taking cell images at 0, 1, 3, 5 and 8 hours (Fig. 5a, HeLa S3 MGMT). We observed a continuous decrease in the fluorescence intensity in the cells over time, which was reduced to around 45% of its original intensity after eight hours (Fig. 5b). To validate that the fluorescence decrease was not due to photobleaching of the fluorescent probe, a negative control experiment was conducted by transfecting MGMT-deficient CHO cells with SNAP-H2B plasmid. SNAP-tag protein is a self-labeling protein derived from MGMT that uses the same O^6^-BG pseudosubstrate and follows the same reaction mechanism as MGMT.^40^ Reaction of **BG-CCVJ** with SNAP-tag also gave a similar fluorescence enhancement ratio as MGMT (Fig. S4†). However, cellular alkylated SNAP-tag has been known to resist degradation compared to alkylated-MGMT.^41^ Under the same imaging conditions, no obvious fluorescence reduction was observed for the nucleus localized SNAP-H2B proteins alkylated with **BG-CCVJ** (Fig. 5a, CHO SNAP-H2B). The higher stability of CCVJ-labeled SNAP-tag over MGMT was also confirmed *in vitro* upon proteolysis with trypsin (Fig. S10†).

From the MGMT degradation time course, it was estimated that the half-life of the alkylated-MGMT in the cytosol of the HeLa S3 cells was around 6 hours, which is about 2-times longer than that obtained using Western blot analysis.^38^ It is important to note that the longer half-life obtained in our experiment was due to the fluorescence turn-off mechanism of the probe, which requires the complete degradation of the protein with the unfolding of the O^6^-BG binding site, thereby releasing CCVJ from the state of restricted rotation. In contrast, only partial degradation of the alkylated-MGMT is sufficient to decrease the alkylated-MGMT signal in the Western blot analysis and SDS-PAGE gel analysis. Thus, our **BG-CCVJ** probe provides a new insight into the complete degradation kinetics of alkylated-MGMT in living cells, which cannot be achieved by using traditional analytical methods. By using the **BG-CCVJ** probe, we also studied the degradation of alkylated-MGMT under native conditions. When HeLa S3 cells were treated with temozolomide (DNA alkylating agent) or O^6^-BG (direct alkylation of MGMT in the cytosol) for 14 hours, followed by visualization using **BG-CCVJ**, the mean fluorescence intensity in the cytosol was reduced to 30% compared to the untreated cells (Fig. S11†). This result supports the previously proposed mechanism for MGMT degradation in HeLa S3 cells, where active MGMT is localized in the cytosol and transported into the nucleus after the protein commences the repair reaction in the nucleus.^38,42,43^

### Fluorescence-switching probe **SA-CCVJ** for the detection of endogenous hCAII

**Fig. 6:**
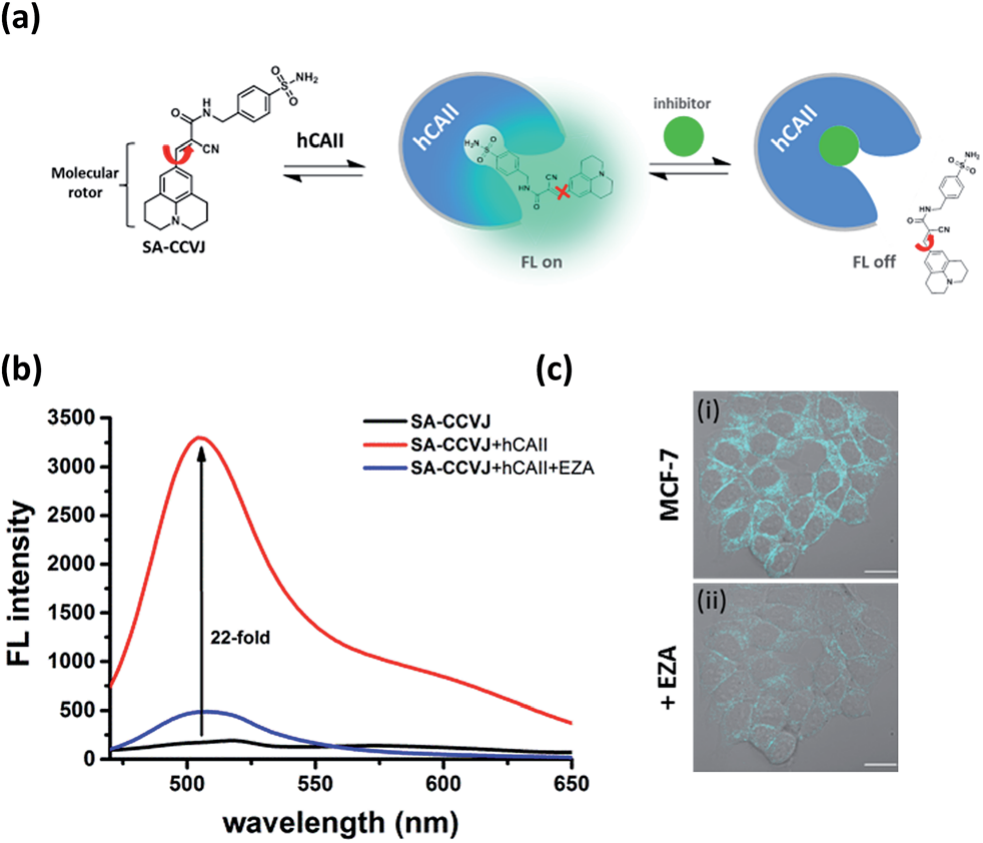
Fluorescence-switching probe **SA-CCVJ** for the detection of endogenous hCAII in living cells. (a) Schematic illustration of the dynamic fluorescence-switchable probe **SA-CCVJ** for the detection of hCAII and in the presence of the sulfonamide drug. (b) Fluorescence spectra of 2 μM **SA-CCVJ** in the absence and presence of 2 μM hCAII in PBS buffer (1% DMSO) and after the addition of 100 μM ethoxzolamide (EZA). (c) DIC and fluorescence overlaid images of living MCF-7 cells upon treatment with (i) 0.5 μM **SA-CCVJ** and (ii) after the addition of 100 μM ethoxzolamide. All cellular images were taken with an identical microscope setup. Scale bar: 20 μm.

To demonstrate the modular nature of our fluorescent probe design, we conjugated the CCVJ dye with benzenesulfonamide to generate **SA-CCVJ** for the fluorescence activation detection of human carbonic anhydrase II (hCAII).^44^ Unlike the interaction of **BG-CCVJ** with MGMT, which involves the formation of a covalent bond, the detection of hCAII by **SA-CCVJ** involving the benzenesulfonamide site is non-covalent and requires a non-enzymatic process. In the presence of hCAII, we expect that the fluorescence intensity should increase due to the steric effects restricting the intramolecular bond rotation. On the other hand, the fluorescence intensity should reduce upon the addition of the sulfonamide drug competing for the binding domain (Fig. 6a). hCAII and many of its isoforms are important proteins in the regulation of numerous physiological process, including pH and CO_2_ homeostasis, bone resorption, calcification, and tumorigenicity.^45^

Similar to the MGMT probe **BG-CCVJ**, the **SA-CCVJ** probe displayed a very weak fluorescence signal in PBS buffer. Upon the addition of hCAII protein, the fluorescence intensity was enhanced by 22-fold (Fig. 6b). The enhancement was reduced dramatically when the hCAII competitive inhibitor ethoxzolamide (100 μM) was added. This result shows that the fluorescence signal of **SA-CCVJ** is dynamically switchable and regulated by the specific binding of the benzenesulfonamide ligand with hCAII. From the hCAII titration experiment, the LOD and *K*_d_ were determined to be around 5 nM and 70 nM, respectively (Fig. S12†). The specificity of **SA-CCVJ** was also investigated by incubating **SA-CCVJ** with eight other non-target proteins and a fluorescence intensity increase was observed only when hCAII was present (Fig. S13†). The fluorescence enhancement mechanism of **SA-CCVJ** in the presence of hCAII should be similar to that of **BG-CCVJ**, as it only exhibits a strong emission in viscous glycerol solution among the tested solvents (Fig. S14†).

In fluorescence live-cell imaging, it is essential to remove the background fluorescence before protein visualization by performing a washing step to remove the unbound fluorescent probe. However, this washout procedure may also wash the highly cell permeable probe away from the protein, resulting in weaker fluorescence signals. This problem is more severe if the binding affinity of the ligand with the protein involves weak non-covalent interactions.

To demonstrate that our fluorescence-switchable probe design can be employed to overcome the limitations of fluorescent probes to image intracellular proteins, no-wash imaging of cytosolic hCAII was then conducted in live MCF-7 cells, which naturally express hCAII.^13^ When 0.5 μM **SA-CCVJ** was added to live MCF-7 cells and incubated at 37 °C for one hour, a strong fluorescence signal was observed in the cytosol region (Fig. 6c). To validate that the strong fluorescence signal was due to the specific interaction of **SA-CCVJ** with hCAII, 100 μM ethoxzolamide was subsequently added, which resulted in a dramatic reduction in the fluorescence intensity in the same cells. In contrast, the addition of the DMSO control to the **SA-CCVJ** treated MCF-7 cells did not result in a decrease in the fluorescence intensity (Fig. S15†). This no-wash operation is very critical for the visualization of intracellular hCAII, as washing of the MCF-7 cells after one hour of incubation with **SA-CCVJ** resulted in a dramatic decrease in the fluorescence intensity in the cells (Fig. S16†). These results indicate that a no-wash operation is essential for fluorescent probes to image intracellular proteins that exhibit a high cell permeability and non-covalent interactions with the target protein.

## Conclusion

In conclusion, we have successfully developed a general design for fluorescence-switchable probes that can be applied for the analysis of endogenous protein expression levels and their degradation status in living cells, as demonstrated by the detection of MGMT and hCAII. The design is based on the conjugate of a fluorescent molecular rotor, CCVJ, and a protein binding ligand. The probe produces a strong fluorescence signal upon interaction with its target protein due to the restricted rotation of the fluorescent molecular rotor. Compared to the existing analytical methods, our probe can be prepared *via* simple synthetic steps and it exhibits a remarkable sensitivity and selectivity for the detection of MGMT and hCAII. Although MGMT fluorescent probes can also be constructed using fluorescent dyes, such as bodipy or rhodamine, conjugated to an O^6^-BG pseudosubstrate, extensive washing to remove these probes is necessary as they exhibit a high background fluorescence signal.^39^ Furthermore, real-time tracking of alkylated-MGMT degradation at the single cell level is not possible with these non-fluorescence off/on probes. Based on the same design, the hCAII probe **SA-CCVJ** was constructed and applied for the imaging of intracellular hCAII proteins in MCF-7 cells. Finally, we believe that our highly versatile and modular design for fluorescence-switchable probes can possibly be extended for the detection of other proteins, for which there are still no effective fluorescence activation probes to image them in living cells.
