# Supplementary material for: Protein sensing in living cells by molecular rotor-based fluorescence-switchable chemical probes
Source: Chem Sci. 2015 Oct 1;7(1):301–7. doi: 10.1039/c5sc02808f (PMC5515057; doi:10.1039/c5sc02808f)
Supplement: Supplementary file 1 [file SC-007-C5SC02808F-s001.pdf]

Electronic Supplementary Material (ESI) for Chemical Science.
This journal is © The Royal Society of Chemistry 2015

SUPPORTING INFORMATION

Protein Sensing in Living Cells by Molecular Rotor-Based Fluorescence Switchable Chemical Probes

Wan-Ting Yu,[a] Ting-Wei Wu,[a] Chi-Ling Huang,[a] I-Chia Chen,[a,b] and Kui-Thong Tan[a,b,]*

a) Department of Chemistry, National Tsing Hua University, 101 Sec. 2, Kuang Fu Rd, Hsinchu 30013, Taiwan (ROC)

b) Frontier Research Center on Fundamental and Applied Sciences of Matters, National Tsing Hua University, 101 Sec. 2, Kuang Fu Rd, Hsinchu 30013, Taiwan (ROC)

Corresponding author: Kui-Thong Tan, Email: kttan@mx.nthu.edu.tw

Materials and instruments

Chemicals and reagents were purchased from Sigma-Aldrich and TCI and were used without further purification. All solvents were used after appropriate distillation or purification. Besides the MGMT, SNAP-tag and hCAII proteins which were expressed and purified in our laboratory, all other proteins used in the selectivity test were purchased from Sigma-Aldrich. PBS buffer (0.9 mM KCl, 2.67 mM KH_2PO_4, 138 mM NaCl, 8.1 mM Na_2HPO_4) was diluted 10-times from commercially available concentrates supplied by Amersco. Thin layer chromatography (TLC) was performed on TLC-aluminum sheets (Silica gel 60 F254, Merck). Flash column chromatography was performed with silica gel (230-400 mesh, Merck). HPLC analysis was performed with analytical column (EC 150/4.6 Nucleosil 300-5 C18, Macherey-Nagel). Products were purified by semi-preparative column (VP 150/21 Nucleosil 300-5 C18, Macherey-Nagel). Anti-MGMT antibody (sc-166528) and anti-α-tubulin antibody were obtained from Santa Cruz Biotechnology (Santa Cruz, CA, USA), respectively. Anti-mouse IgG was obtained from GE Healthcare Life Sciences. $pSNAP_f$-H2B was purchased from New England Biolab (MA, USA).

^{1}H, and ^{13}C nuclear magnetic resonance (NMR) spectra were recorded on Bruker DMX-400 and Mercury-400 with ^{1}H chemical shifts (δ) reported in ppm relative to the solvent residual signals of $CDCl_3$ (7.24 ppm), CD_3OD (3.30 ppm), d-DMSO (2.49 ppm). ^{13}C chemical shifts (δ) were reported in ppm relative to the solvent residual signals of d-DMSO (39.5 ppm). Coupling constants were reported in Hz. Absorption spectra were recorded on Hitachi U-3310 spectrophotometer. Fluorescence spectra were recorded using Hitachi F-4500 fluorescence spectrophotometer and TECAN Infinite M200Pro. High resolution mass spectra (HRMS) were recorded on Varian 901-FTMS. In-gel fluorescence was carried out by using Ettan DIGE imager (GE healthcare).

MGMT and SNAP-tag protein expression and purification

Plasmids pET51b-MGMT, pET51b-SNAP-tag and pET51b-hCAII with C-terminal His-tag were transformed to *E. coli* strain BL21. The bacteria was cultured at 37 °C in LB broth containing 100 µg/mL ampicillin to OD_{600} of 1.2. Protein expression was induced by the addition of 1 mM IPTG. After 16 h at 18 °C, the cultures were harvested by centrifugation. The cells were lysed by sonication and insoluble protein and cell debris were removed by centrifugation. The MGMT and SNAP-tag proteins were then purified by Ni-NTA. The purified proteins were concentrated and transferred in PBS buffer using Amicon® Ultra centrifugal filters. The proteins were snap frozen in liquid nitrogen before being stored at -78 °C. Concentration of the proteins was determined using BCA assay. Purity of the proteins was checked by SDS-PAGE and stained either by Instant Blue or Coomassie Blue. (MGMT: 26 kD, SNAP-tag: 23 kD)

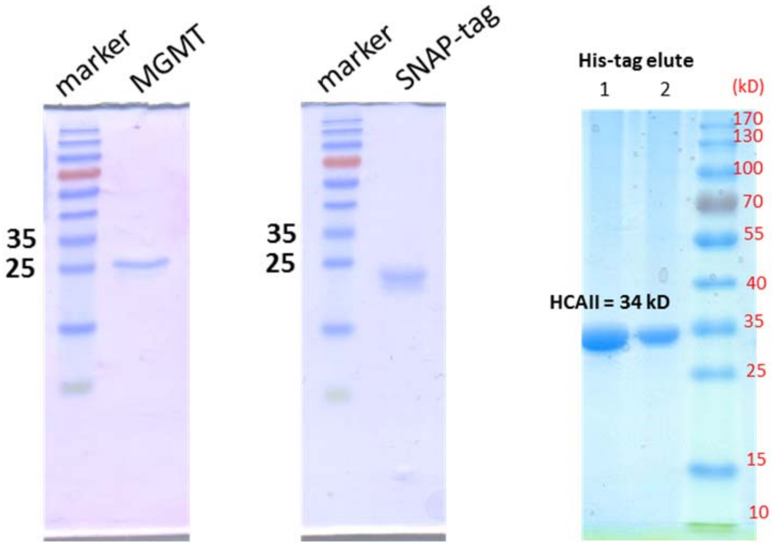

Polypeptide sequence of recombinant MGMT protein

```
MASWSHPQFE   KGADDDDKVP   MDKDCEMKRT   TLDSPLGKLE   LSGCEQGLHE
                          >>------------------------MGMT---------------------------------->
IKLLGKGTSA   ADAVEVPAPA   AVLGGPEPLM   QCTAWLNAYF   HQPEAIEEFP
>-----------------------------------------------------MGMT----------------------------------------------------->
VPALHHPVFQ   QESFTRQVLW   KLLKVVKFGE   VISYQQLAAL   AGNPKAARAV
>-----------------------------------------------------MGMT----------------------------------------------------->
GGAMRGNPVP   ILIPCHRVVC   SSGAVGNYSG   GLAVKEWLLA   HEGHRLGKPG
>-----------------------------------------------------MGMT----------------------------------------------------->
LGGSSGLAGA   WLKGAGATS    GSPPAGRNRA   PGFSSISAHH   HHHHHHHH
>-------------------------MGMT----------------------------->>                   >>------His-tag------>>
```

Polypeptide sequence of recombinant SNAP-tag protein

```
MASWSHPQFE   KGADDDDKVP   MDKDCEMKRT   TLDSPLGKLE   LSGCEQGLHE
                          >>--------------------------SNAP--------------------------------->
IIFLGKGTSA   ADAVEVPAPA   AVLGGPEPLM   QATAWLNAYF   HQPEAIEEFP
>-------------------------------------------------------SNAP------------------------------------------------------->
VPALHHPVFQ   QESFTRQVLW   KLLKVVKFGE   VISYSHLAAL   AGNPAATAAV
>-------------------------------------------------------SNAP------------------------------------------------------->
KTALSGNPVP   ILIPCHRVVQ   GDLDVGGYEG   GLAVKEWLLA   HEGHRLGKPG
>-------------------------------------------------------SNAP------------------------------------------------------->
LGTSRAPGFS   SISAHHHHHH   HHHH
->>                     >>------His-tag------>>
```

Polypeptide sequence of recombinant hCAII protein

```
MASWSHPQFE   KGADDDDKVP   AGGMSHHWGY   GKHNGPEHWH   KDFPIAKGER
                               >>----------------hCAII--------------------------------->
QSPVDIDTHT   AKYDPSLKPL   SVSYDQATSL   RILNNGHAFN   VEFDDSQDKA
>-----------------------------------------------------hCAII------------------------------------------------------>
VLKGGPLDGT   YRLIQFHFHW   GSLDGQGSEH   TVDKKKYAAE   LHLVHWNTKY
>-----------------------------------------------------hCAII------------------------------------------------------>
GDFGKAVQQP   DGLAVLGIFL   KVGSAKPGLQ   KVVDVLDSIK   TKGKSADFTN
>-----------------------------------------------------hCAII------------------------------------------------------>
FDPRGLLPES   LDYWTYPGSL   TTPPLLECVT   WIVLKEPISV   SSEQVLKFRK
>-----------------------------------------------------hCAII------------------------------------------------------>
LNFNGEGEPE   ELMVDNWRPA   QPLKNRQIKA   SFKRAPGFSS   ISAHHHHHHH
>-------------------------------hCAII------------------------------------>>              >>--His-tag-->
HHH
--->>
```

Cell culture

Hela S3 and HEK293 cells were cultured in DMEM medium supplemented with 10% fetal bovine serum and 1% penicillin-streptomycin. MCF-7 cells were cultured in MEM medium supplemented as above. CHO cells were cultured in F-12K medium supplemented as above. All cultures were incubated at 37 °C under a humidified atmosphere of 95% air and 5% CO_2.

No-wash fluorescence imaging of MGMT levels with BG-CCVJ in different cell lines

Cells were maintained in a culture medium supplemented with 10% FBS and 1% penicillin-streptomycin. 1.5×10^4 cells were seeded in 8-well chamber slides and cultured overnight at 37 °C in air with 5% CO_2. Cells were washed twice with Opti-MEM and 1 μM **BG-CCVJ** probe in Opti-MEM (1.0 % DMSO (v/v)) was added. After incubation for 90 minutes at 37 °C in air with 5% CO_2, cell images were taken without removing the excess probe by using Laser Scanning Confocal Microscope (LSM 700, Zeiss, Germany). The images were taken using a 405 nm laser and a BP 490-555 emission filter.

CHO Cells transfected with pSNAP$_f$-H$_2$B

CHO cells were maintained in F-12K supplemented with 10% FBS and 1% penicillin-streptomycin. 1.5×10^4 cells were seeded in 8-well chamber slides and cultured overnight at 37 °C in air with 5% CO_2. The cells were then transfected by using X-treme GENE HP DNA transfection reagent (Roche Applied Science) according to the manufacturer's protocol. Thirty hours after transfection, the cells were washed with F-12K supplemented with 10% FBS twice and then cultured for another twelve hours before imaging.

Western blot

Cell extracts were prepared by washing cells with PBS buffer and solubilizing 1×10^7 cells in 300 µl of lysis buffer (50 mM Tris, 100 mM NaCl, 1 mM DTT, pH 7.5). The cells were lysed with freeze-thaw methods for three cycles. The lysates were centrifuged at 12,000 rpm for 30 min at 4 °C and the supernatant was collected. 16 µl/lane of cell lysate was loaded to 10% SDS-PAGE gel. Following electrophoretic transfer of proteins onto poly(vinyl difluoride) membranes, the membranes were blocked with 5% nonfat dry milk in PBST buffer (138 mM NaCl, 2.68 mM KCl, 1.76 mM KH_2PO_4, 10.14 mM Na_2HPO_4, 0.5% tween20, pH 7.4). The membranes were washed and then incubated with anti-MGMT antibody (0.2 µg/mL) at 4 °C overnight. After three 10-min wash with PBST buffer, the membranes were incubated with a secondary antibody (anti-mouse IgG; 0.2 µg/mL) for 1 hr. The membranes were washed before being visualized by 4CN PLUS chromogenic substrate (Perkin Elmer).

Detecting MGMT proteins in cell lysates with BG-CCVJ

Cells were maintained in culture medium supplemented with 10% FBS and 1% penicillin-streptomycin at 37 °C in air with 5% CO_2. Cells were washed twice with PBS buffer and were treated with 5 µM **BG-CCVJ** prepared in culture medium (1.0 % DMSO (v/v)). After incubation for 90 min at 37 °C in air with 5% CO_2, cell lysates were prepared by washing cells with PBS buffer (3 x) and solubilizing 1×10^7 cells in 300 µl of lysis buffer (50 mM Tris, 100 mM NaCl, 1 mM DTT, pH 7.5). The cells were lysed with freeze-thaw methods for three cycles. The lysates were centrifuged at 12,000 rpm for 30 min at 4 °C and the supernatant was collected. The supernatant were diluted 6.7-times (5×10^5/100 µL) and the fluorescence intensity was recorded by fluorescence spectrophotometer.

Fluorescence lifetime measurements: For time-resolved measurements, the excitation laser wavelength was 400 nm and the power used was less than 1 mW. The laser system is a femto second mode-locked Ti:sapphire laser generating a pulse train (82 MHz, 800 nm) of which the second-harmonic pulses are generated with a nonlinear crystals (BBO, type I). Picosecond time-resolved fluorescence was measured by time-correlated single-photon counting (TCSPC). The samples were placed in a cuvette, and the fluorescence was filtered through a bandpass filter (±10 nm) and detected by a multichannel plate photomultiplier (MCP-PMT, Hamamatsu). The instrument response function was set at 40 ps at fwhm. The polarization of laser beam was kept at the magic angle with respect to the detection position. We obtained the time constants from the experimental curves by deconvoluting a biexponential function against the instrument response function with fwhm ~40 ps (assuming Gaussian function).

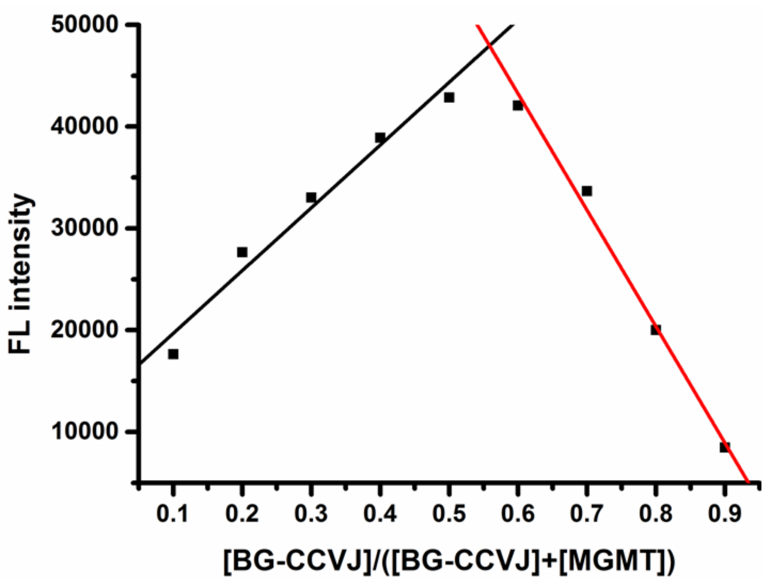

Figure S1. Job's plot analysis of probe **BG-CCVJ** with MGMT.

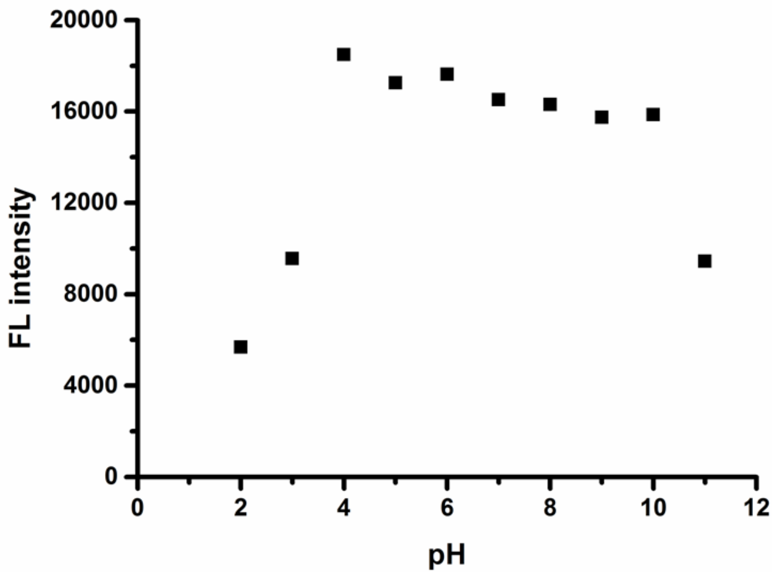

Figure S2. Reaction of **BG-CCVJ** with MGMT protein at different pH. Dramatic fluorescence increase can be obtained for **BG-CCVJ** and MGMT incubated within pH 4-10.

(a) (b)

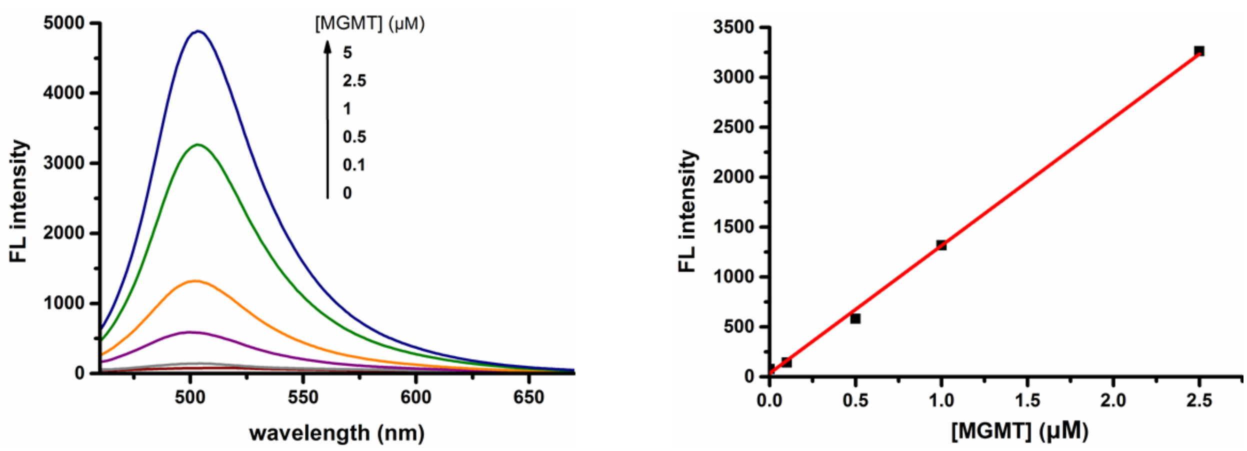

Figure S3. (a) Fluorescence spectra for the addition of increasing MGMT concentration to 5 μM **BG-CCVJ**. (b) Fluorescence response was linear in the range of 0-2.5 μM MGMT. The LOD is about 5 nM.

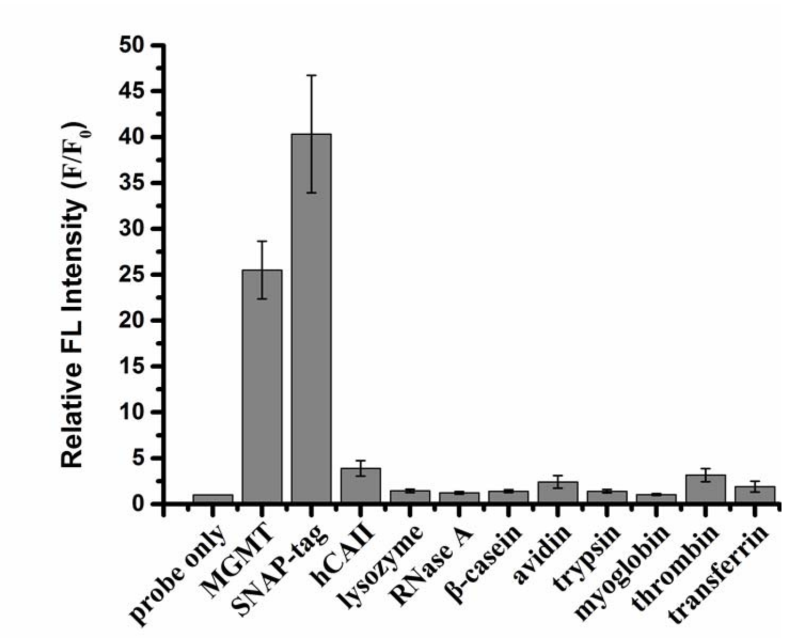

Figure S4. Selectivity test of 5 μM **BG-CCVJ** with MGMT, SNAP-tag and nine other non-targeted proteins (all in 1 μM). hCAII = human carbonic anhydrase II, RNAase A = Ribonuclease A. Error bars were calculated from three independent measurements.

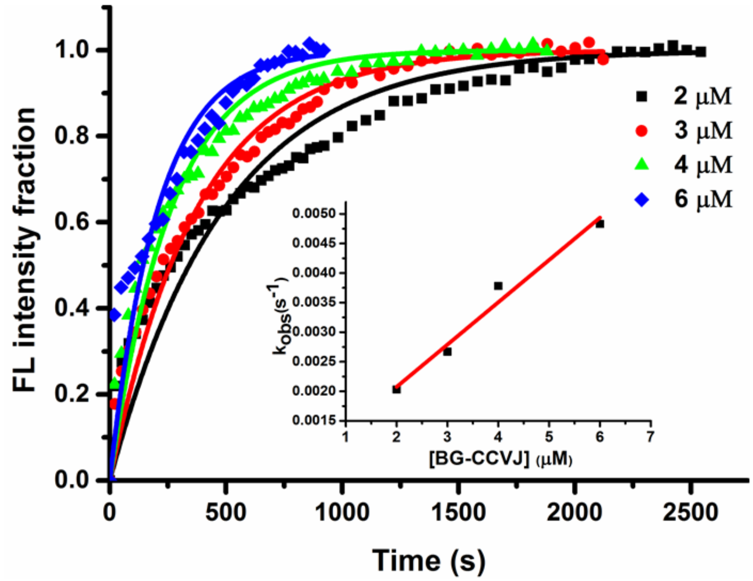

Figure S5. Fluorescence increase of **BG-CCVJ** (2 – 6 μM) in the presence of MGMT (100 nM). The inset shows the linear relationship plot of probe concentration versus calculated k^{obs} (R^2 = 0.98). The second-order rate constant (k_2) for the reaction between **BG-CCVJ** and MGMT was determined to be about 715 $M^{-1}s^{-1}$.

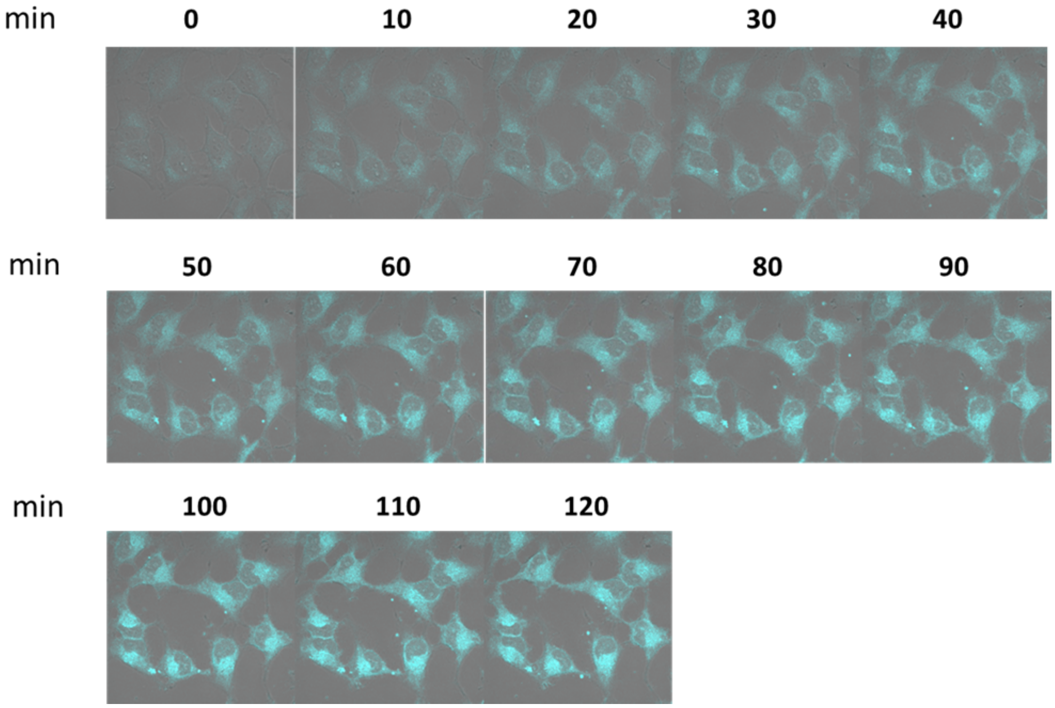

Figure S6. Fluorescence time-course of Hela S3 cells treated with 1 μM **BG-CCVJ**. After 100 minutes of incubation, the fluorescence reached its maximum signal. The result is consistent with the in vitro labeling rate data which reached maximum fluorescence after 90 minute incubation with MGMT protein.

(a)

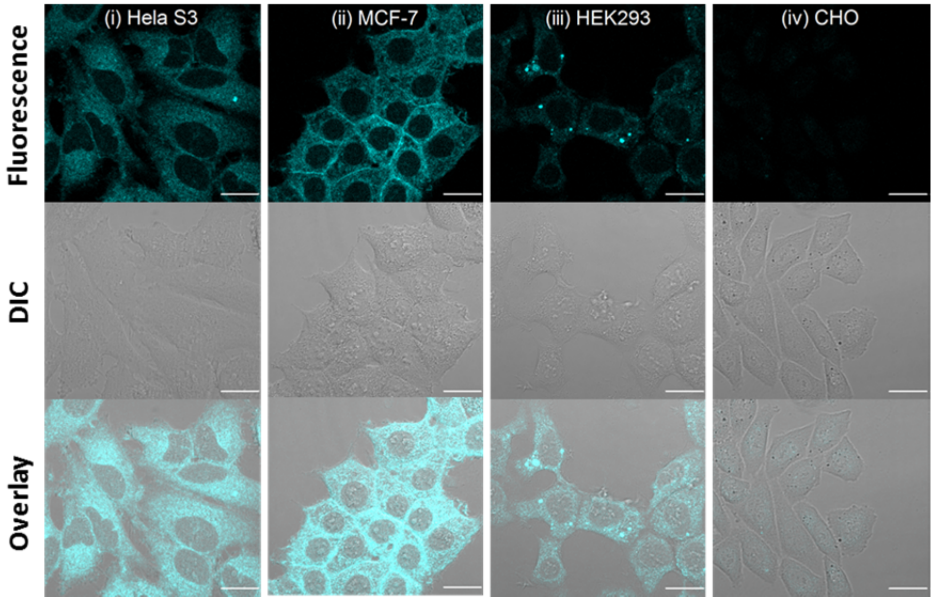

(b)

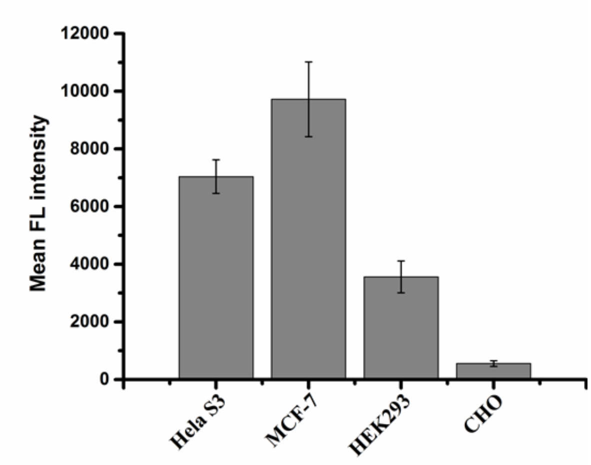

Figure S7. Live-cell imaging of MGMT activity with **BG-CCVJ** in different cell lines with washing operations. (a) Images of live Hela S3, MCF-7, HEK293 and CHO cells treated with 1 μM **BG-CCVJ**. Images were taken with three times washing with Opti-MEM after 90 minutes of incubation with **BG-CCVJ**. All cellular images were taken with identical microscope setup. Scale bar: 20 μm. (b) Mean fluorescence intensity of the cells treated with **BG-CCVJ** (N = 20). The mean fluorescence intensity of each cell was calculated pixel-by-pixel using ImageJ software.

(a)

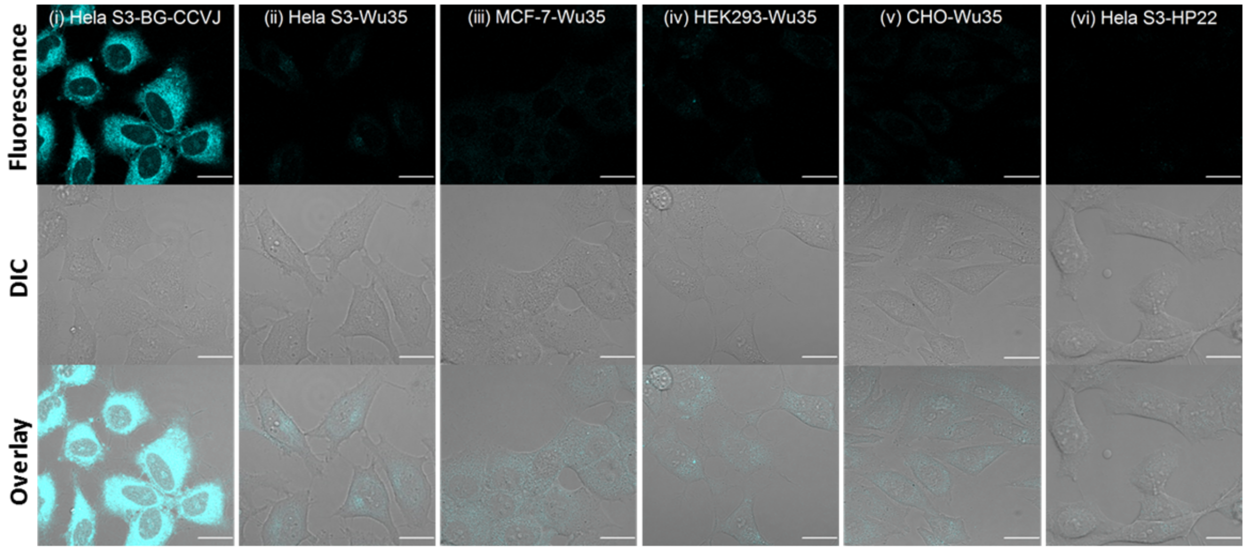

(b)

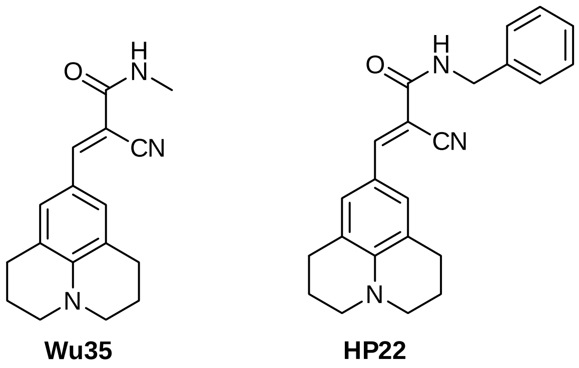

Figure S8. (a) Images of Hela S3, MCF-7, HEK293 and CHO cells treated with 1 μM negative-control compounds **Wu35** and **HP22.** Hela S3 cells treated with **BG-CCVJ** was included for comparison. The images were taken immediately without washing procedures after 90 minutes of incubation. All cellular images were taken with identical microscope setup. Scale bar: 20 μm. (b) Chemical structures of **Wu35** and **HP22**.

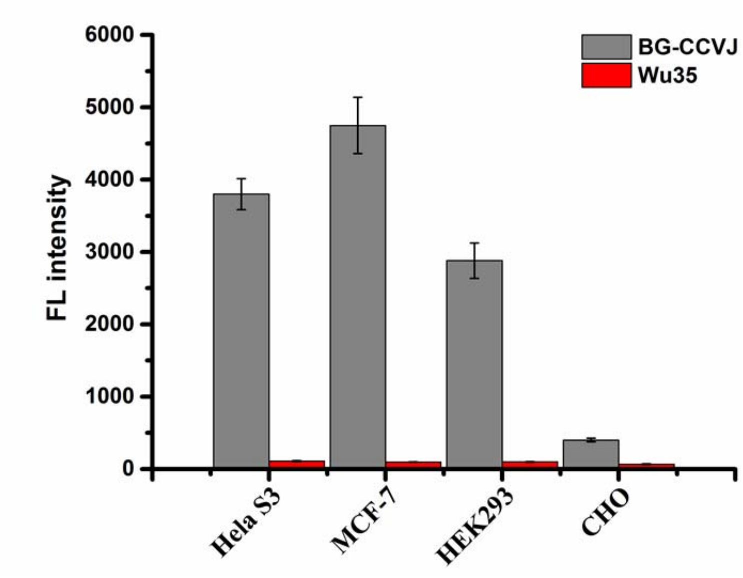

Figure S9. Quantification of MGMT proteins in the cell lysates of Hela S3, MCF-7, HEK293 and CHO with **BG-CCVJ**. The results showed that **BG-CCVJ** can be used to distinguish cells which expressed different levels of MGMT. The negative-control **Wu35** (without the O^6-benzylguanine moiety) displayed very weak fluorescence for the four cell lines. Error bars were calculated from three independent measurements.

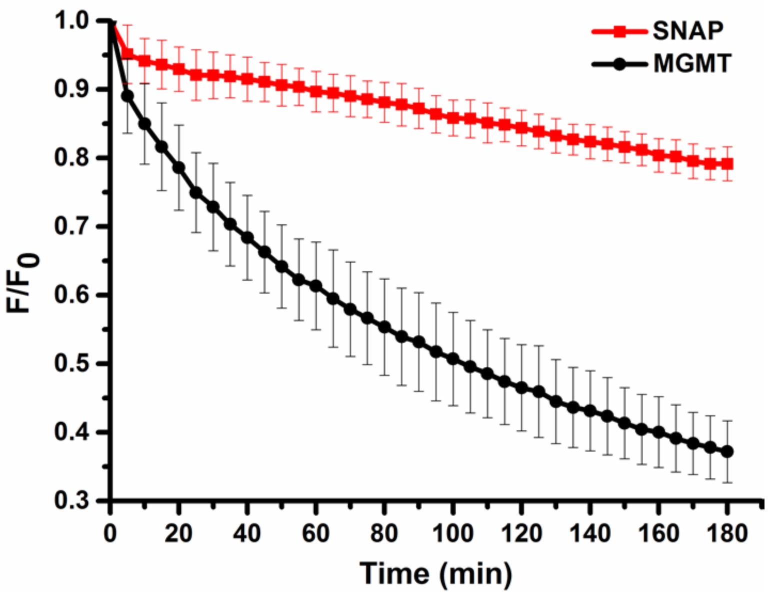

Figure S10. Time course of the 5 μM CCVJ-labeled MGMT and SNAP-tag protein degradation under proteolysis condition (5 μM trypsin). Emission was monitored continuously at 504 nm. Error bars were calculated from three independent measurements.

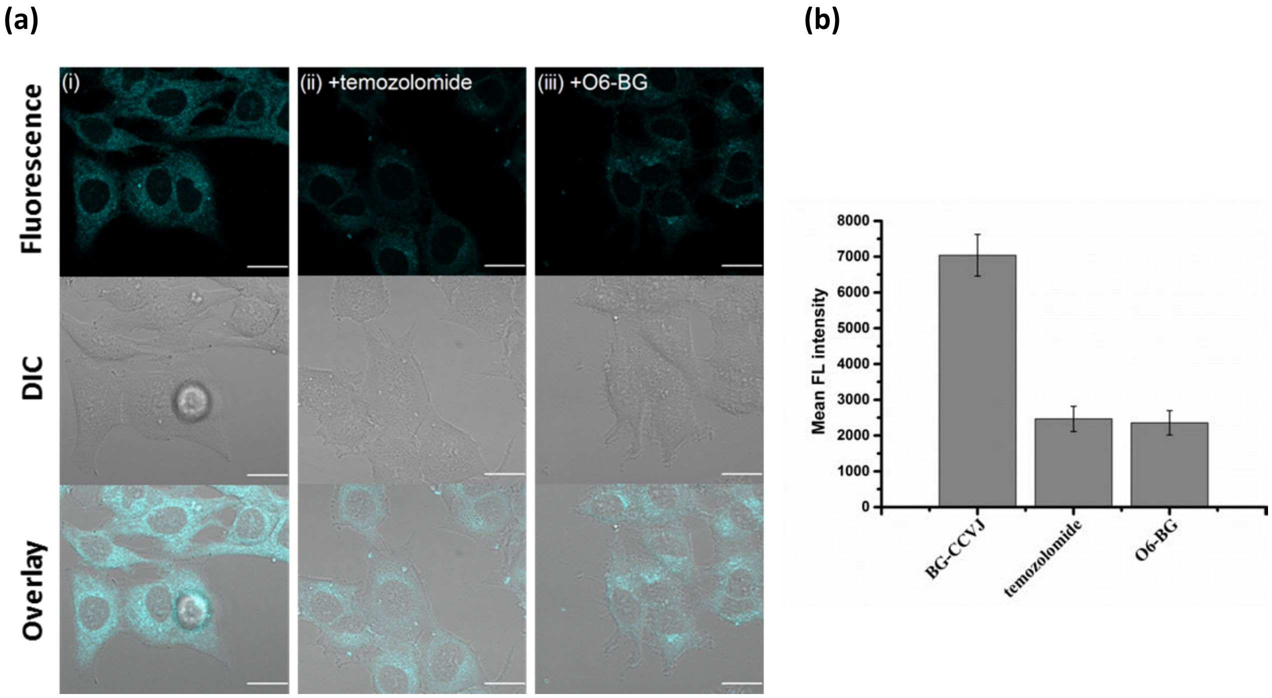

Figure S11. Temozolomide and O^6-BG induced MGMT degradation in living Hela S3 cells investigated by **BG-CCVJ**. (a) Hela S3 cells treated with (i) 1 μM **BG-CCVJ** only, (ii) 100 μM temozolomide for 14 hours followed by 1 μM **BG-CCVJ**, and (iii) 50 μM O6-BG for 14 hours followed by 1 μM **BG-CCVJ**. The cells were washed before imaging. All cellular images were taken on the same day with identical microscope setup. Scale bar: 20 μm. (b) Mean fluorescence intensity of Hela S3 cells incubated with or without temozolomide and O^6-BG (N = 20).

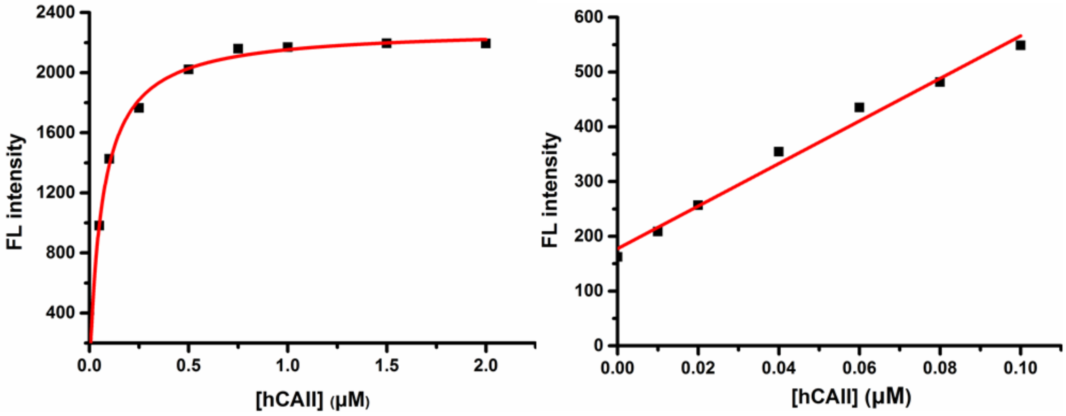

Figure S12. (a) Titration curve of 0.5 **SA-CCVJ** with increasing hCAII concentrations. The data was fitted to one site binding equation to obtain the K_d of about 70 nM. (b) Fluorescence response of increasing hCAII concentrations to 2 μM **SA-CCVJ**. The response was linear in the range of 0-0.1 μM hCAII. The LOD is about 5 nM.

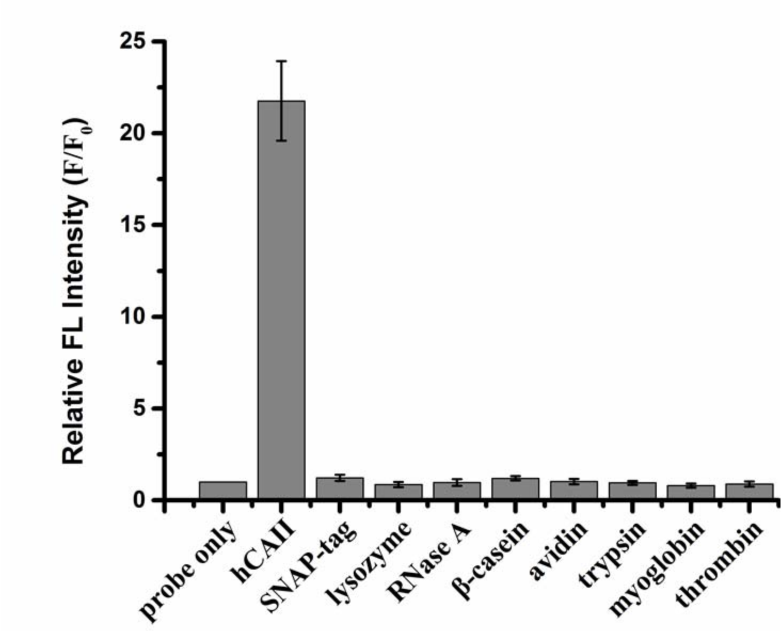

Figure S13. Selectivity test of 2 μM **SA-CCVJ** with hCAII and eight other non-targeted proteins (all in 2 μM). A. Error bars were calculated from three independent measurements.

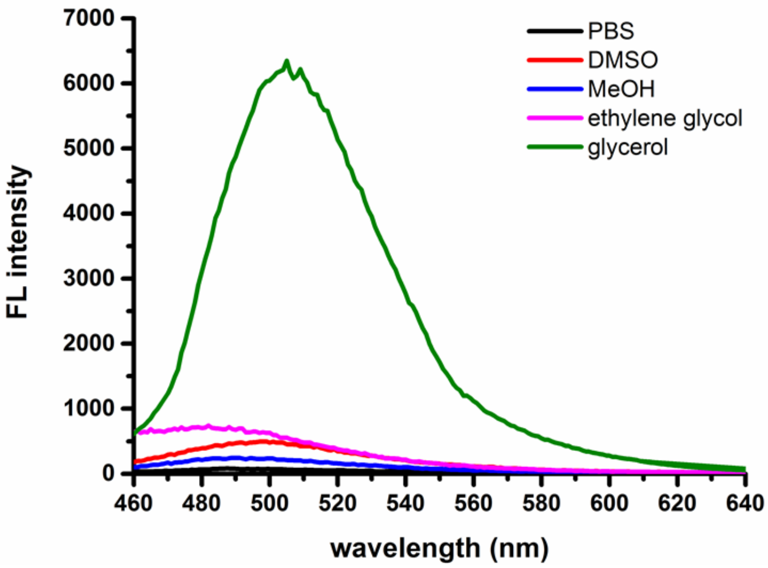

Figure S14. Emission spectra of 5 μM probe **SA-CCVJ** in DMSO, MeOH, ethylene glycol, PBS and glycerol.

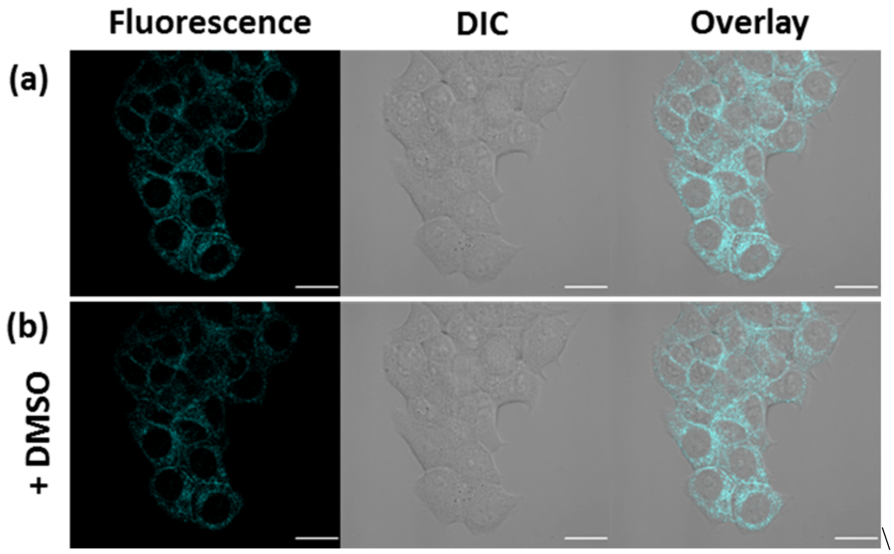

Figure S15. Images of living MCF-7 cells upon treatment with (a) 0.5 μM **SA-CCVJ** and (b) after addition of DMSO control. All cellular images were taken with identical microscope setup. Scale bar: 20 μm.

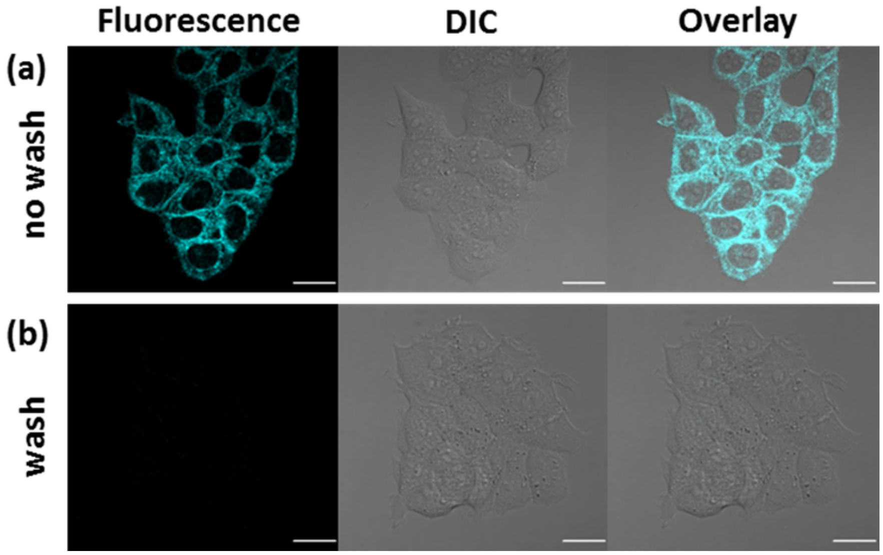

Figure S16. After treatment with 1 μM **SA-CCVJ**, images of living MCF-7 cells were taken under (a) no-wash and (b) washing operations. All cellular images were taken with identical microscope setup. Scale bar: 20 μm.

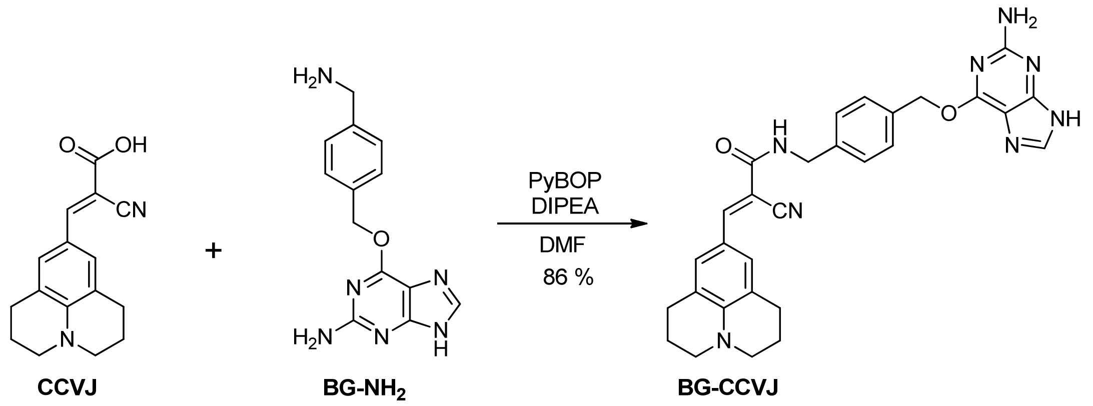

Scheme S1. Synthesis of **BG-CCVJ**

Synthesis of BG-CCVJ

To a 5 mL reaction flask containing CCVJ (30 mg, 0.11 mmol), BG-NH_2 (19 mg, 0.07 mmol), and PyBOP (36.4 mg, 0.14 mmol) in DMF was added DIPEA (0.35 mmole) at room temperature. The reaction mixture was stirred at room temperature overnight. The solvent was removed and the crude mixture was purified by reversed-phase preparative HPLC to give product **BG-CCVJ** as an orange solid in 86% yield. **^{1}H NMR** (400 MHz, DMSO) δ 8.55 (t, *J* = 5.8 Hz, 1H), 7.83 (s, 1H), 7.79 (s, 1H), 7.44 (d, *J* = 7.9 Hz, 2H), 7.41 (s, 2H), 7.30 (d, *J* = 7.9 Hz, 2H), 6.27 (s, 2H), 5.44 (s, 2H), 4.37 (d, *J* = 5.8 Hz, 2H), 3.31-3.25 (m, 4H), 2.66-2.63 (m, 4H), 1.89-1.81 (m, 4H) ppm; **^{13}C NMR** (100 MHz, DMSO) δ 162.53, 159.85, 159.62, 155.18, 150.40, 146.64, 139.28, 137.76, 135.30, 130.23, 128.44, 127.41, 120.39, 118.53, 117.62, 113.51, 94.49, 66.50, 49.30, 42.85, 27.05, 20.63 ppm; **HRMS** (ESI): m/z calc. for $C_{29}H_{29}N_8O_2$ $[M+H]^+$ 521.2406, found 521.2408 $[M+H]^+$.

CCVJ and BG-NH_2 were prepared as previously reported:

(1) C. Rumble, K. Rich, G. He, M. Maroncelli, *J. Phys. Chem. A.* **2012**, *116*, 10786–10792.

(2) A. Keppler, S. Gendreizig, T. Gronemeyer, H. Pick, H. Vogel, K. Johnsson, *Nat. Biotechnol.* **2003**, *21*, 86.

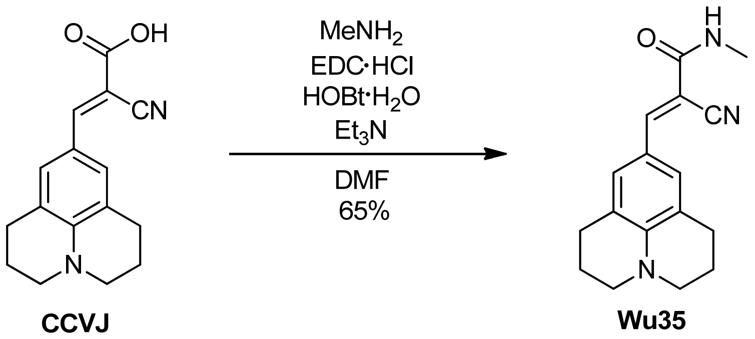

Scheme S2. Synthesis of **Wu35**

Synthesis of Wu35

To a 5 mL reaction flask containing CCVJ (10 mg, 37 µmol), EDC·HCl (21.44 mg, 112 µmol), HOBt·H_2O (17.15 mg, 112 µmol) and Et_3N (15.61 µL, 112 µmol) in DMF was added 40% $MeNH_2$ in methanol (4.45 µL, 45 µmol) at room temperature. The reaction mixture was stirred at room temperature overnight. The crude product was extracted with ethyl acetate and 1 M HCl (3x each) and the organic phase was washed three times with brine. The organic phase was dried with $MgSO_4$, filtered and concentrated in vacuo. The crude residue was purified by column chromatography using ethyl acetate and n-hexane (1:5) as the eluent to afford **Wu35** as an orange solid. Yield: 65%. **^{1}H NMR** (400 MHz, $CDCl_3$) δ 8.00 (s, 1H), 7.42 (s, 2H), 3.38-3.18 (m, 4H), 2.94 (d, *J* = 4.8 Hz, 3H), 2.83-2.60 (m, 4H), 2.05-1.82 (m, 4H) ppm. **^{13}C NMR** (100 MHz, $CDCl_3$) δ 163.19, 152.26, 147.00, 130.97, 120.71, 119.55, 118.55, 93.25, 50.06, 27.58, 26.98, 21.17 ppm. **HRMS** (ESI): m/z calc. for $C_{17}H_{20}N_3O$ $[M+H]^+$ 282.1606, found 282.1601 $[M+H]^+$.

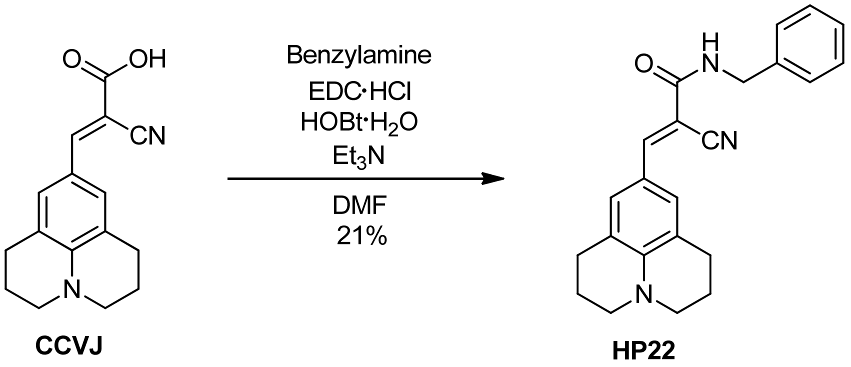

Scheme S3. Synthesis of **HP22**

Synthesis of HP22

To a 5 mL reaction flask containing CCVJ (20 mg, 0.075 mmol), EDC·HCl (28.76 mg, 0.15 mmol), HOBt·H_2O (22.97 mg, 0.15 mmol) and Et_3N (104.5 µL, 0.75 mmol) in DMF was added benzylamine (40.4 µL, 0.37 mmol) at room temperature. The reaction mixture was stirred at room temperature overnight. The solvent was removed and the crude mixture was purified by reversed-phase preparative HPLC to give product **HP22**. Yield: 21%. **^{1}H NMR** (400 MHz, CD_3OD) δ 8.23 (s, 1H), 7.56-7.18 (m, 7H), 4.80 (s, 2H), 3.52-3.38 (m, 4H), 2.79-2.70 (m, 4H), 1.99-1.93 (m, 4H) ppm. **^{13}C NMR** (100 MHz, $CDCl_3$) δ 162.58, 152.70, 147.13, 137.84, 131.08, 128.73, 127.83, 127.58, 120.72, 119.43, 118.57, 92.99, 50.07, 44.29, 27.57, 21.15 ppm. **HRMS** (ESI): m/z calc. for $C_{23}H_{24}N_3O$ $[M+H]^+$ 358.1919, found 358.1915 $[M+H]^+$.

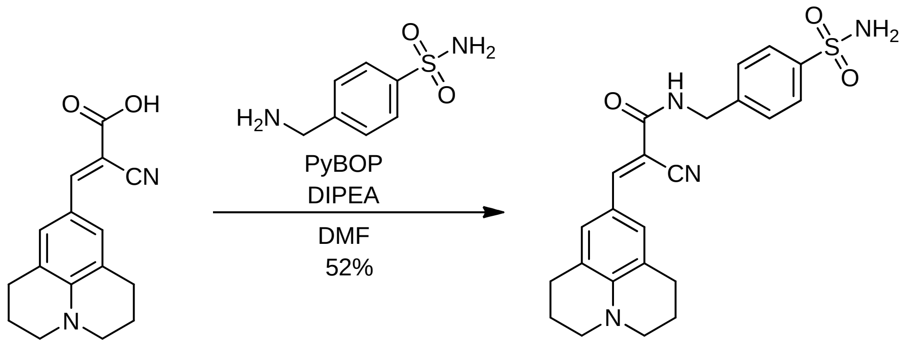

Scheme S4. Synthesis of **SA-CCVJ**

Synthesis of SA-CCVJ

To a 5 mL reaction flask containing CCVJ (30 mg, 0.11 mmol), 4-(Aminomethyl)-benzensulfonamide (38 mg, 0.17 mmol), and PyBOP (117 mg, 0.22 mmol) in DMF was added DIPEA (0.56 mmole) at room temperature. The reaction mixture was stirred at room temperature for 2 h. The solvent was removed and the crude mixture was purified by reversed-phase preparative HPLC to give product **SA-CCVJ** as a yellow solid in 52% yield. **^{1}H NMR** (400 MHz, DMSO) δ 8.63 (t, *J* = 5.9 Hz, 1H), 7.84 (s, 1H), 7.76 (d, *J* = 8.0 Hz, 2H), 7.45 (d, *J* = 8.0 Hz, 2H), 7.42 (s, 2H), 7.30 (s, 2H), 4.43 (d, *J* = 5.9 Hz, 2H), 3.30-3.25 (m, 4H), 2.68-2.62 (m, 4H), 1.90-1.80 (m, 4H). **^{13}C NMR** (100 MHz, DMSO) δ 162.65, 150.58, 146.71, 143.54, 142.58, 130.29, 127.61, 125.64, 120.40, 118.52, 117.57, 94.11, 49.30, 42.75, 27.04, 20.61. **HRMS** (ESI): m/z calc. for $C_{23}H_{25}N_4O_3S$ $[M+H]^+$ 437.1647, found 437.1643 $[M+H]^+$.

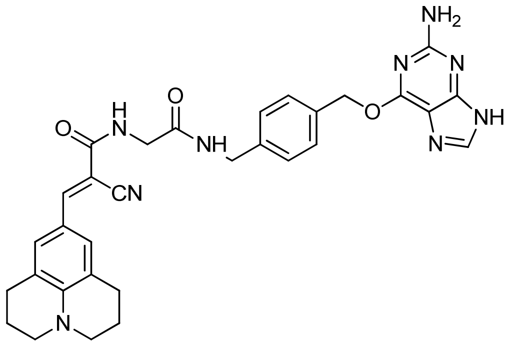

BG-Gly-CCVJ

Based on the synthetic procedure of **BG-CCVJ, BG-Gly-CCVJ** was prepared in 30% yield (10 mg)**.** **^{1}H NMR** (400 MHz, DMSO) δ 8.46 (t, *J* = 5.7 Hz, 1H), 8.12 (t, *J* = 5.8 Hz, 1H), 7.84 (s, 1H), 7.48 (d, *J* = 8.1 Hz, 2H), 7.43 (s, 2H), 7.29 (d, *J* = 8.1 Hz, 2H), 5.50 (s, 2H), 4.29 (d, *J* = 5.8 Hz, 2H), 3.82 (d, *J* = 5.7 Hz, 2H), 3.32-3.27 (m, 4H), 2.70-2.62 (m, 4H), 1.91-1.81 (m, 4H) ppm. **^{13}C NMR** (100 MHz, DMSO) δ 168.72, 162.70, 158.91, 150.48, 146.74, 139.76, 134.22, 130.27, 128.79, 127.28, 120.43, 119.88, 118.64, 117.55, 94.15, 67.83, 49.32, 43.03, 41.84, 27.07, 20.62 ppm; **HRMS** (ESI): m/z calc. for $C_{31}H_{32}N_9O_3$ $[M+H]^+$ 578.2628, found 578.2623 $[M+H]^+$.

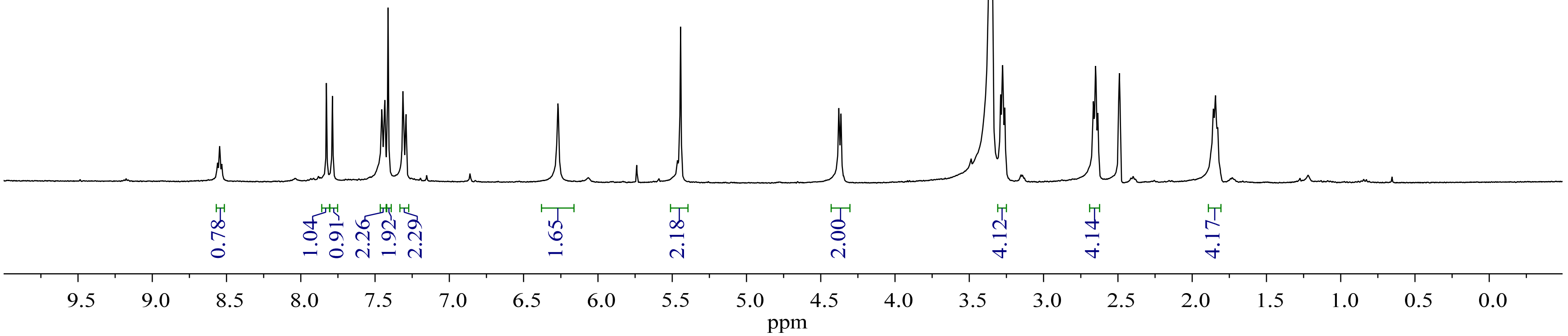

	Parameter	Value
1	Title	YU33
2	Origin	UXNMR, Bruker Analytische Messtechnik GmbH
3	Spectrometer	spect
4	Solvent	DMSO
5	Temperature	300.0
6	Pulse Sequence	zg30
7	Experiment	1D
8	Number of Scans	23
9	Receiver Gain	181
10	Relaxation Delay	2.0000
11	Pulse Width	10.0000
12	Acquisition Time	2.5560
13	Acquisition Date	2014-09-09T14:28:00
14	Modification Date	2014-09-09T14:29:52
15	Spectrometer Frequency	400.15
16	Spectral Width	6410.3
17	Lowest Frequency	-411.3
18	Nucleus	1H
19	Acquired Size	16384
20	Spectral Size	16384

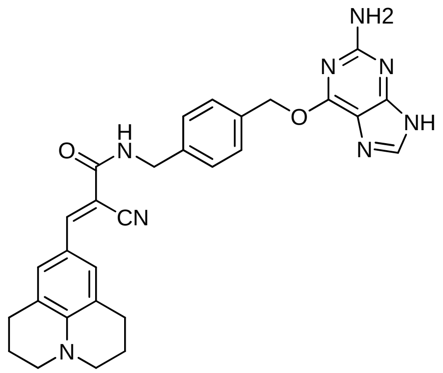

BG-CCVJ

BG-CCVJ_13C

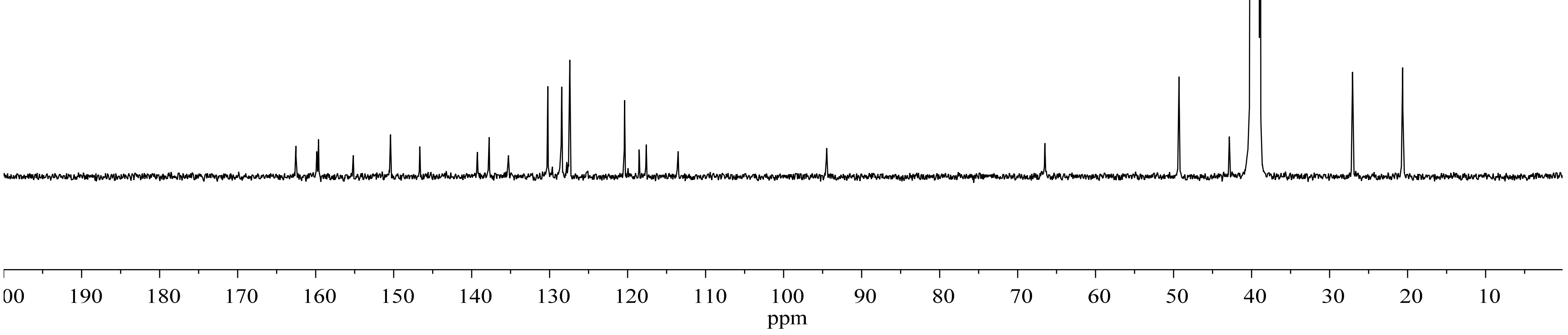

	Parameter	Value
1	Title	YU33
2	Origin	UXNMR, Bruker Analytische Messtechnik GmbH
3	Spectrometer	spect
4	Solvent	DMSO
5	Temperature	300.0
6	Pulse Sequence	zgpg30
7	Experiment	1D
8	Number of Scans	8908
9	Receiver Gain	90
10	Relaxation Delay	2.0000
11	Pulse Width	9.7000
12	Acquisition Time	1.4418
13	Acquisition Date	2014-09-11T00:15:00
14	Modification Date	2014-09-11T08:55:32
15	Spectrometer Frequency	100.62
16	Spectral Width	22727.3
17	Lowest Frequency	-345.2
18	Nucleus	13C
19	Acquired Size	32768
20	Spectral Size	32768

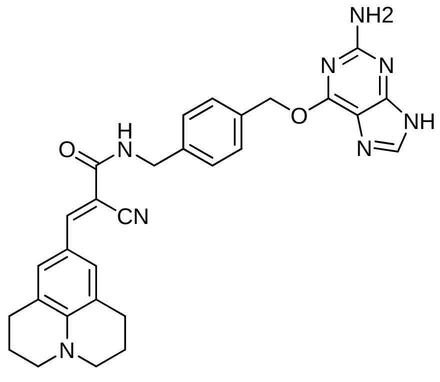

BG-CCVJ

Wu35_1H

Parameter	Value
1 Title	20150528-1
2 Origin	UXNMR, Bruker Analytische Messtechnik GmbH
3 Spectrometer	spect
4 Solvent	CDCl3
5 Temperature	300.0
6 Pulse Sequence	zg30
7 Experiment	1D
8 Number of Scans	9
9 Receiver Gain	406.0
10 Relaxation Delay	2.0000
11 Pulse Width	10.0000
12 Acquisition Time	2.5560
13 Acquisition Date	2015-05-28T13:30:00
14 Modification Date	2015-05-28T13:31:34
15 Spectrometer Frequency	400.15
16 Spectral Width	6410.3
17 Lowest Frequency	-421.4
18 Nucleus	1H
19 Acquired Size	16384
20 Spectral Size	16384

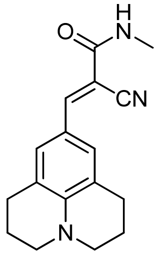

Wu35

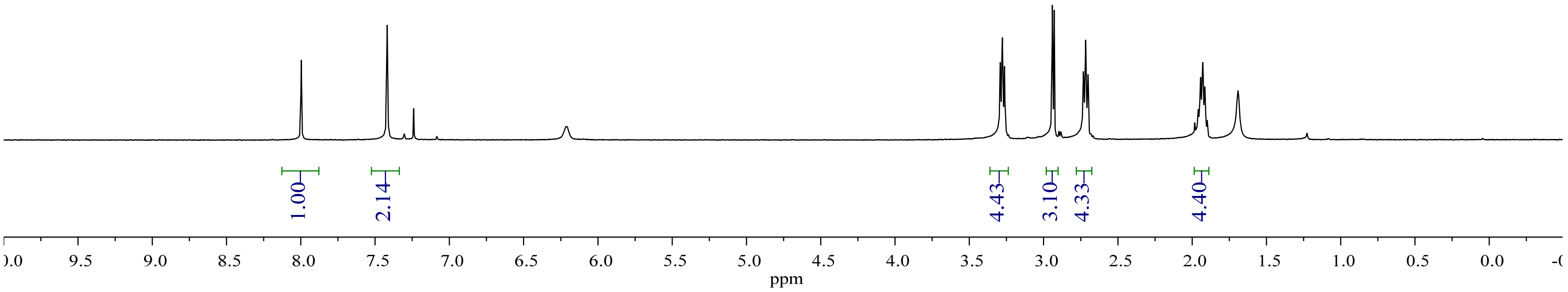

Wu35_13C

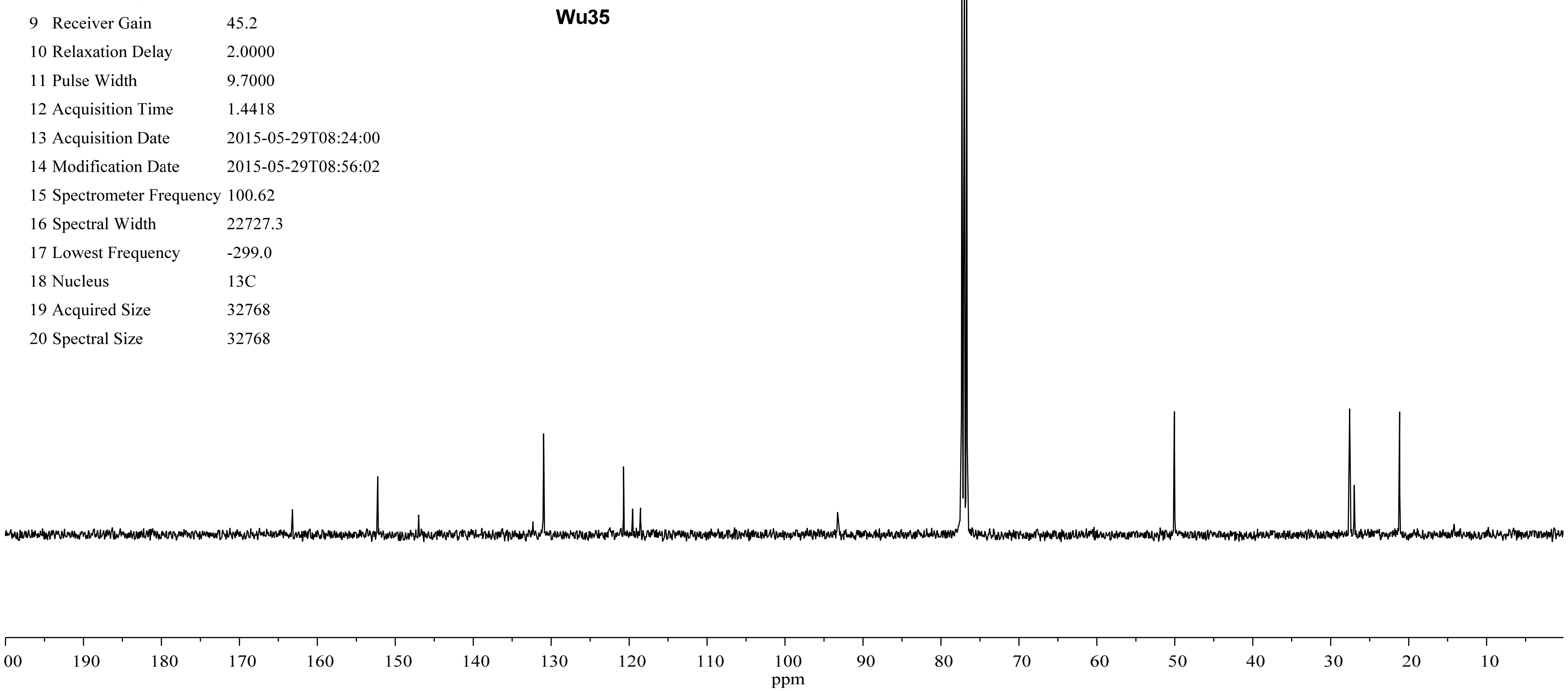

	Parameter	Value
1	Title	20150529-2
2	Origin	UXNMR, Bruker Analytische Messtechnik GmbH
3	Spectrometer	spect
4	Solvent	CDCl3
5	Temperature	300.0
6	Pulse Sequence	zgpg30
7	Experiment	1D
8	Number of Scans	533
9	Receiver Gain	45.2
10	Relaxation Delay	2.0000
11	Pulse Width	9.7000
12	Acquisition Time	1.4418
13	Acquisition Date	2015-05-29T08:24:00
14	Modification Date	2015-05-29T08:56:02
15	Spectrometer Frequency	100.62
16	Spectral Width	22727.3
17	Lowest Frequency	-299.0
18	Nucleus	13C
19	Acquired Size	32768
20	Spectral Size	32768

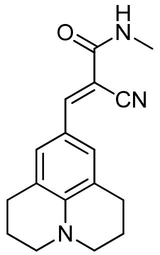

Wu35

HP22_1H

Parameter	Value
1 Title	20150529-3
2 Origin	UXNMR, Bruker Analytische Messtechnik GmbH
3 Spectrometer	spect
4 Solvent	CDCl3
5 Temperature	300.0
6 Pulse Sequence	zg30
7 Experiment	1D
8 Number of Scans	18
9 Receiver Gain	322.0
10 Relaxation Delay	2.0000
11 Pulse Width	10.0000
12 Acquisition Time	2.5560
13 Acquisition Date	2015-05-29T09:12:00
14 Modification Date	2015-05-29T09:14:04
15 Spectrometer Frequency	400.15
16 Spectral Width	6410.3
17 Lowest Frequency	-421.3
18 Nucleus	1H
19 Acquired Size	16384
20 Spectral Size	16384

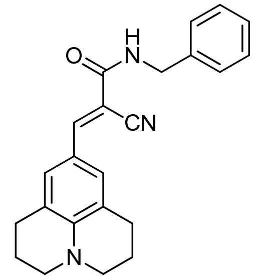

HP22

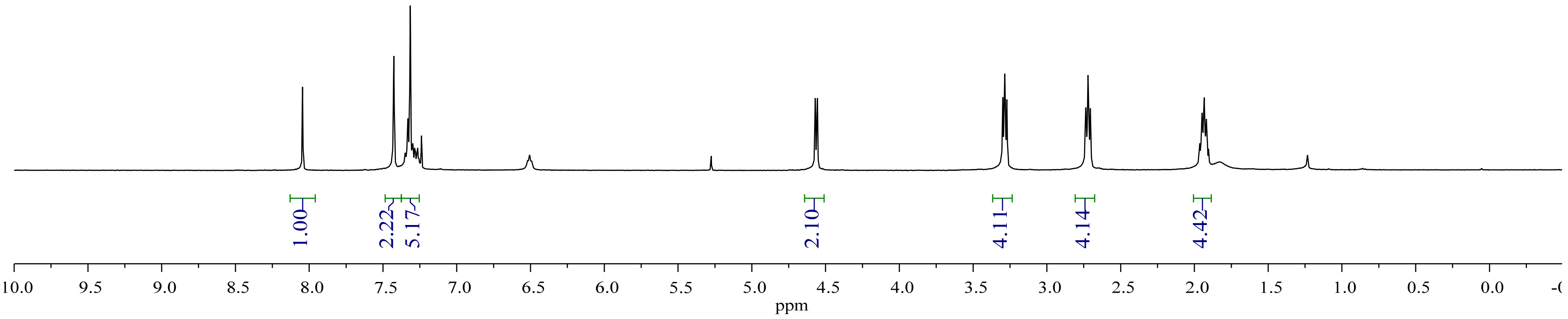

HP22_13C

Parameter	Value
1 Title	20150529-4
2 Origin	UXNMR, Bruker Analytische Messtechnik GmbH
3 Spectrometer	spect
4 Solvent	CDCl3
5 Temperature	300.0
6 Pulse Sequence	zgpg30
7 Experiment	1D
8 Number of Scans	603
9 Receiver Gain	57.0
10 Relaxation Delay	2.0000
11 Pulse Width	9.7000
12 Acquisition Time	1.4418
13 Acquisition Date	2015-05-29T09:16:00
14 Modification Date	2015-05-29T09:52:10
15 Spectrometer Frequency	100.62
16 Spectral Width	22727.3
17 Lowest Frequency	-299.9
18 Nucleus	13C
19 Acquired Size	32768
20 Spectral Size	32768

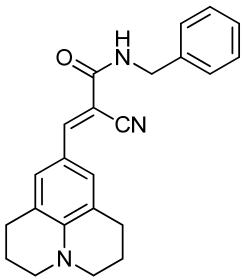

HP22

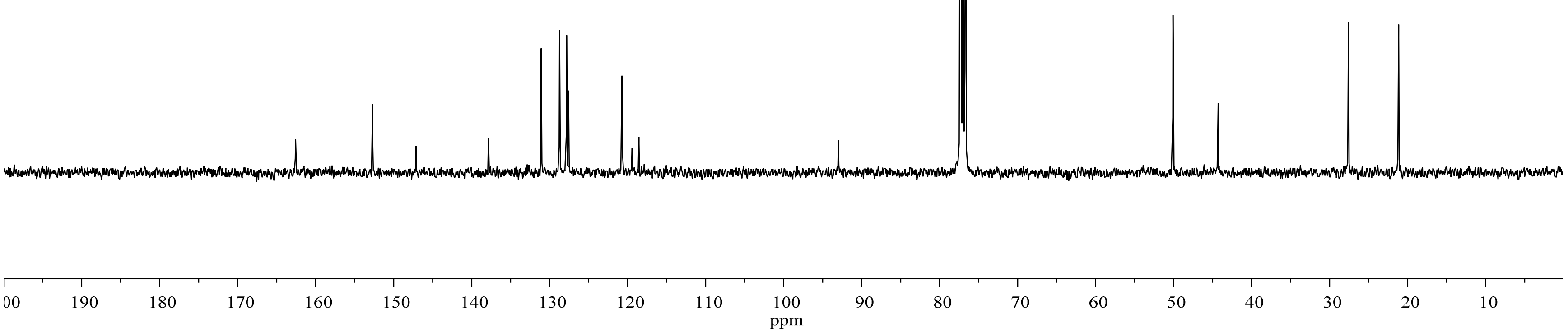

SA-CCVJ_1H

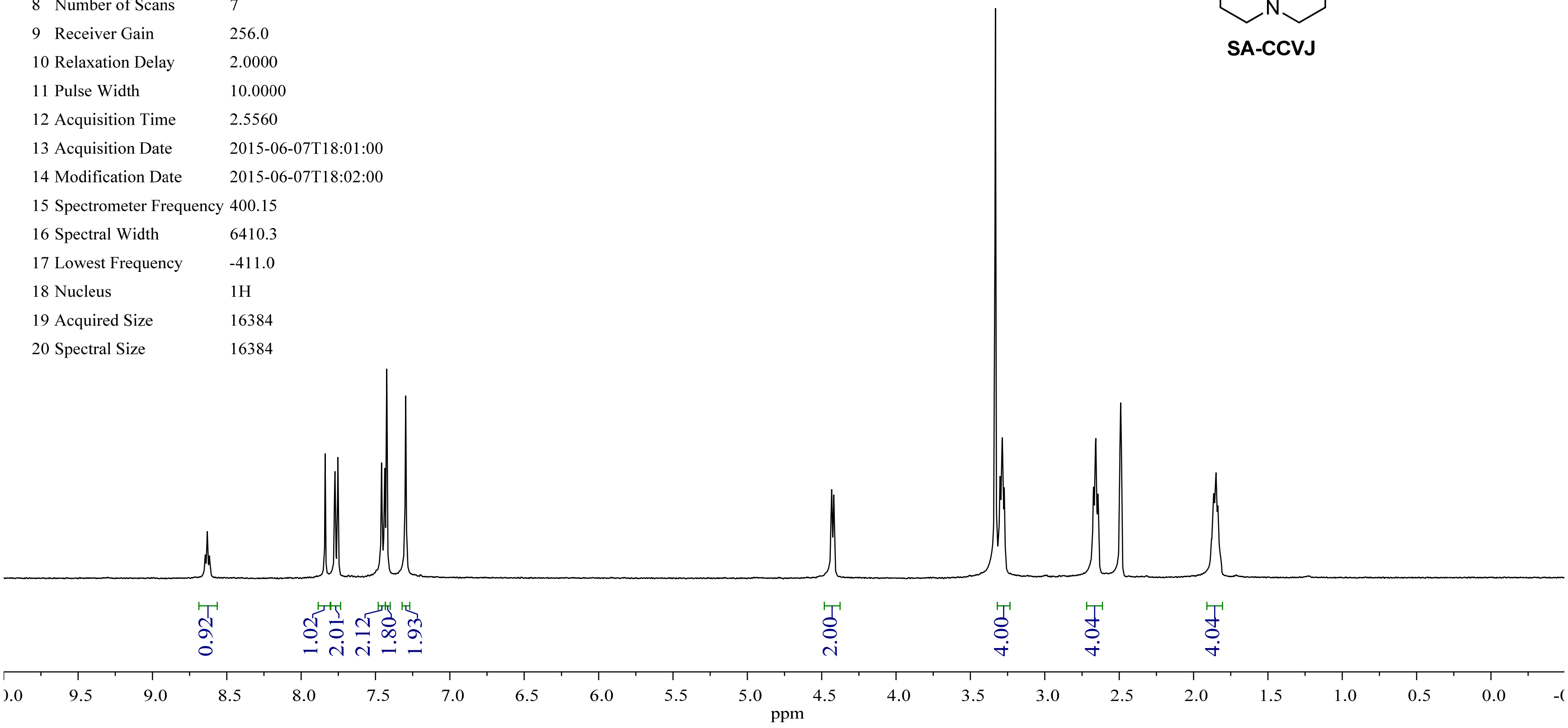

	Parameter	Value
1	Title	20150607-1
2	Origin	UXNMR, Bruker Analytische Messtechnik GmbH
3	Spectrometer	spect
4	Solvent	DMSO
5	Temperature	300.0
6	Pulse Sequence	zg30
7	Experiment	1D
8	Number of Scans	7
9	Receiver Gain	256.0
10	Relaxation Delay	2.0000
11	Pulse Width	10.0000
12	Acquisition Time	2.5560
13	Acquisition Date	2015-06-07T18:01:00
14	Modification Date	2015-06-07T18:02:00
15	Spectrometer Frequency	400.15
16	Spectral Width	6410.3
17	Lowest Frequency	-411.0
18	Nucleus	1H
19	Acquired Size	16384
20	Spectral Size	16384

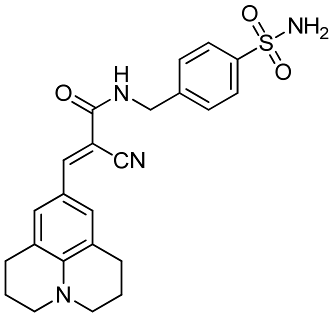

SA-CCVJ

SA-CCVJ_13C

	Parameter	Value
1	Title	20150607-2.2.1.1r
2	Origin	UXNMR, Bruker Analytische Messtechnik GmbH
3	Spectrometer	spect
4	Solvent	DMSO
5	Temperature	300.0
6	Pulse Sequence	zgpg30
7	Experiment	1D
8	Number of Scans	820
9	Receiver Gain	57.0
10	Relaxation Delay	2.0000
11	Pulse Width	9.7000
12	Acquisition Time	1.4418
13	Acquisition Date	2015-06-07T18:03:00
14	Modification Date	2015-06-07T18:51:24
15	Spectrometer Frequency	100.62
16	Spectral Width	22727.3
17	Lowest Frequency	-346.0
18	Nucleus	13C
19	Acquired Size	32768
20	Spectral Size	32768

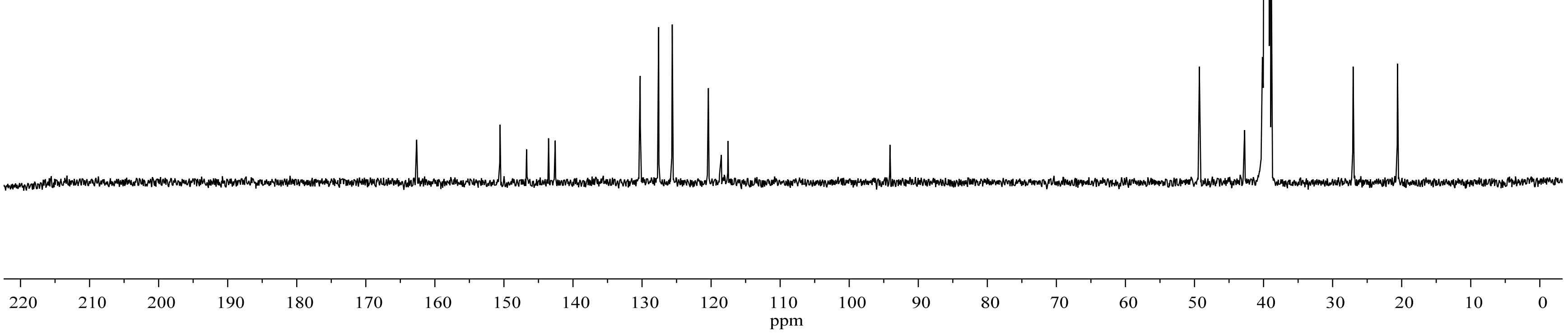

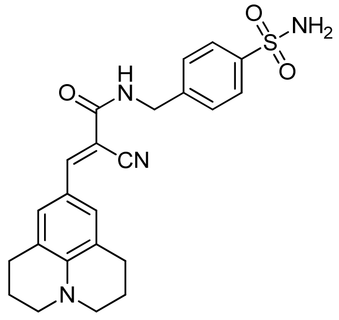

SA-CCVJ

BG-Gly-CCVJ_1H

Parameter	Value
1 Title	TW55-150623
2 Origin	Varian
3 Spectrometer	mercury
4 Solvent	DMSO
5 Temperature	29.0
6 Pulse Sequence	s2pul
7 Experiment	1D
8 Number of Scans	12
9 Receiver Gain	39
10 Relaxation Delay	1.0000
11 Pulse Width	0.0000
12 Acquisition Time	1.9950
13 Acquisition Date	2015-06-23T00:57:25
14 Modification Date	2015-06-23T08:32:00
15 Spectrometer Frequency	400.44
16 Spectral Width	6006.0
17 Lowest Frequency	-986.2
18 Nucleus	1H
19 Acquired Size	11982
20 Spectral Size	32768

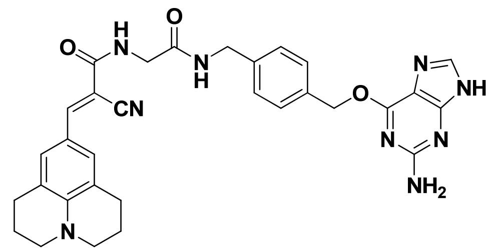

BG-Gly-CCVJ

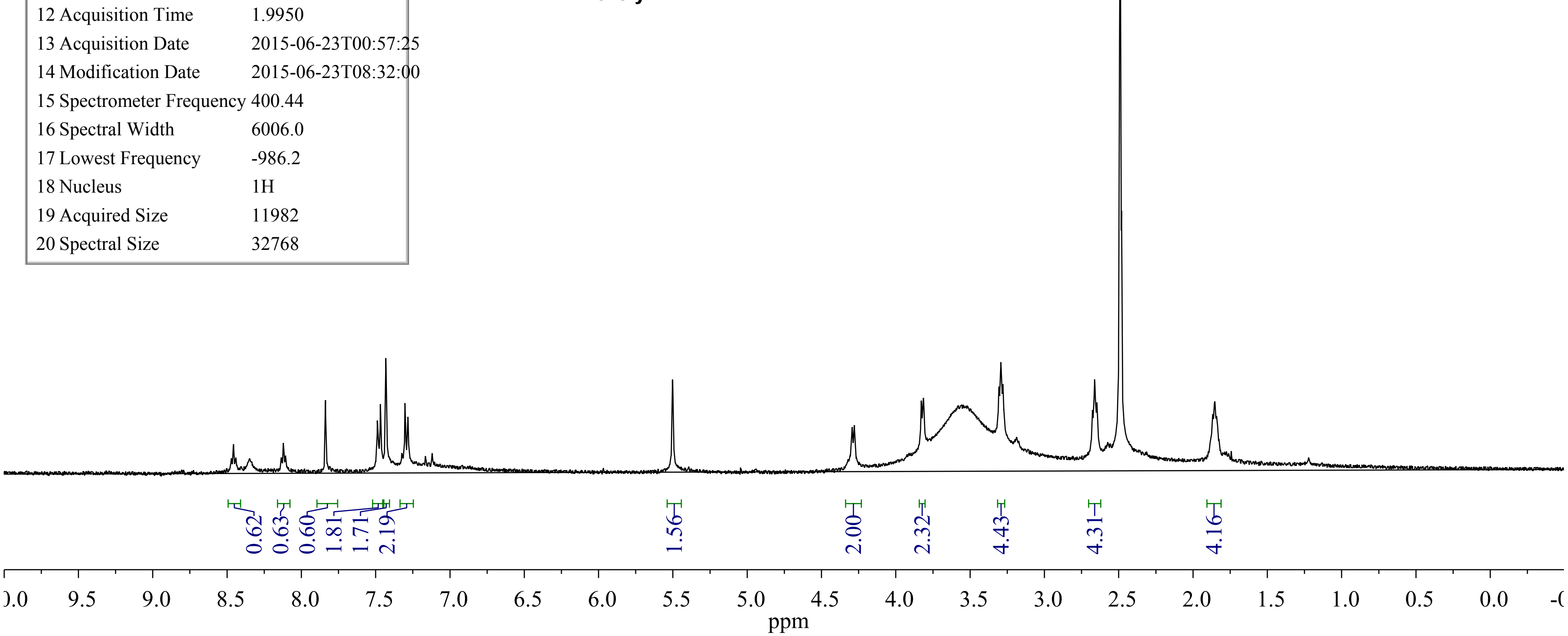

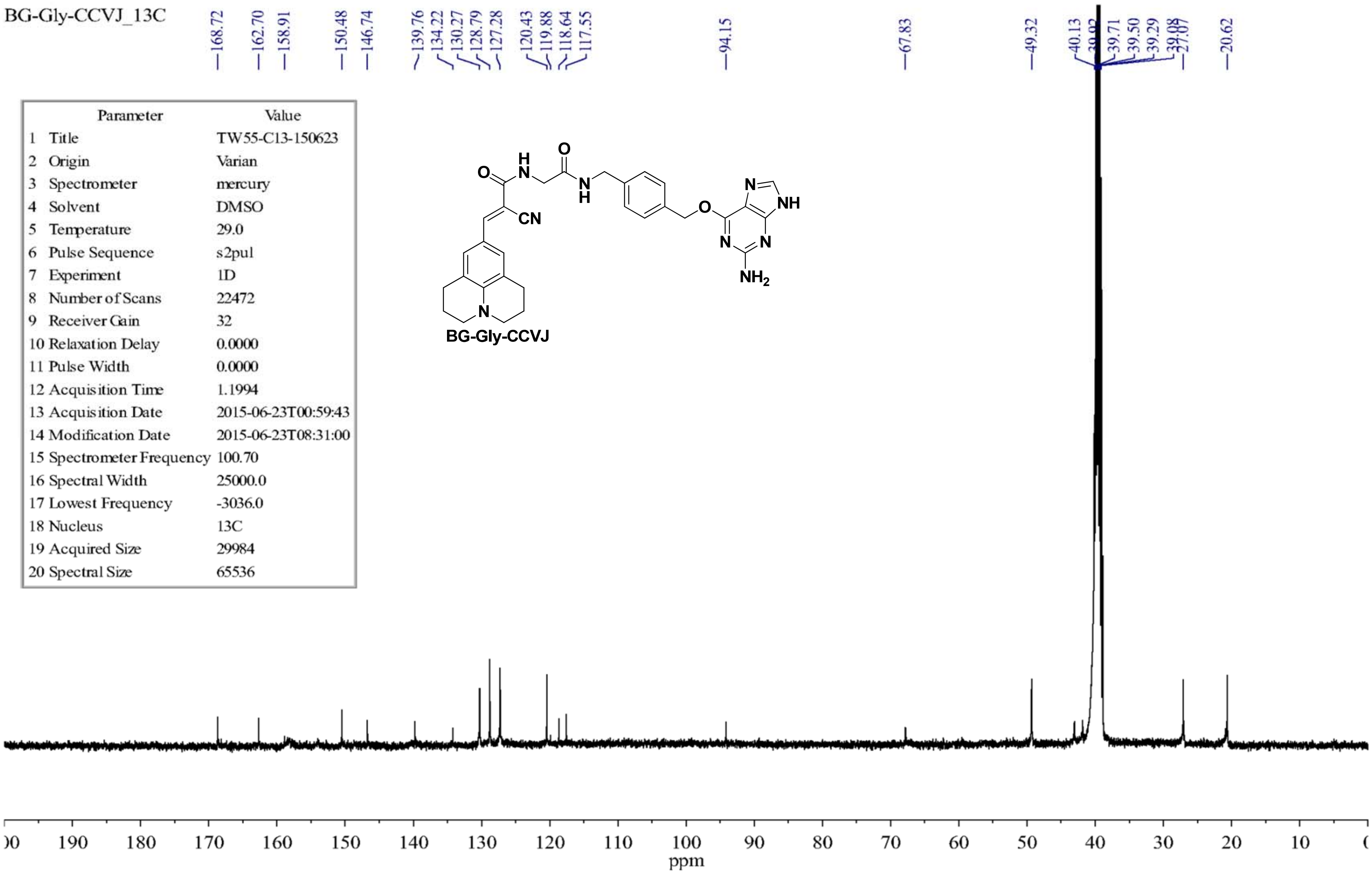

	Parameter	Value
1	Title	TW55-C13-150623
2	Origin	Varian
3	Spectrometer	mercury
4	Solvent	DMSO
5	Temperature	29.0
6	Pulse Sequence	s2pul
7	Experiment	1D
8	Number of Scans	22472
9	Receiver Gain	32
10	Relaxation Delay	0.0000
11	Pulse Width	0.0000
12	Acquisition Time	1.1994
13	Acquisition Date	2015-06-23T00:59:43
14	Modification Date	2015-06-23T08:31:00
15	Spectrometer Frequency	100.70
16	Spectral Width	25000.0
17	Lowest Frequency	-3036.0
18	Nucleus	13C
19	Acquired Size	29984
20	Spectral Size	65536
